# Linear Gold(I) Halide Complexes with a Diamidocarbene Ligand: Synthesis, Reactivity, and Phosphorescence

**DOI:** 10.1021/acs.organomet.3c00360

**Published:** 2023-11-20

**Authors:** Charlotte Riley, Nguyen Le Phuoc, Mikko Linnolahti, Alexander S. Romanov

**Affiliations:** † Department of Chemistry, The University of Manchester, Oxford Road, Manchester M13 9PL, United Kingdom; ‡ Department of Chemistry University of Eastern Finland, FI-80101 Joensuu, Finland

## Abstract

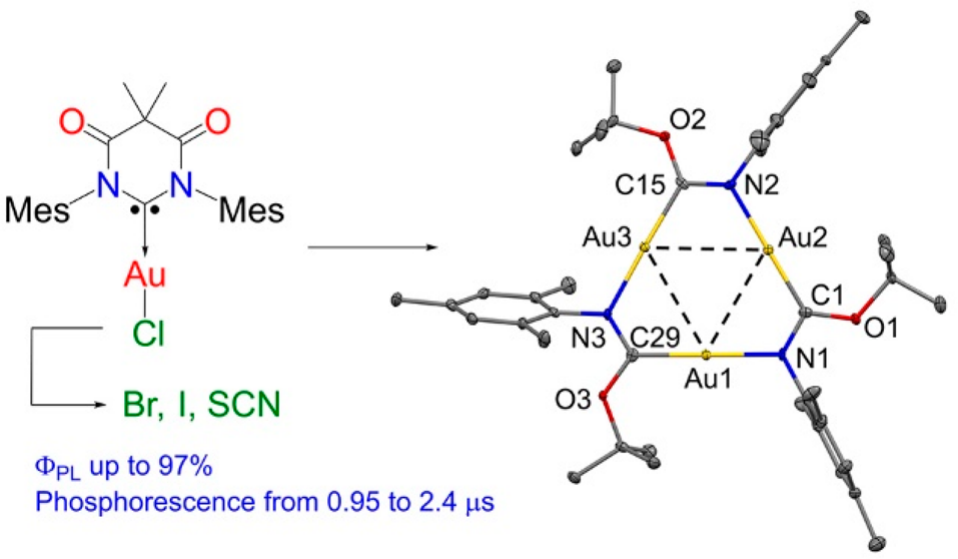

A series of halide and pseudohalide gold complexes (DAC)Au(I)X (DAC = *N*,*N*′-diamidocarbene; X = Cl, Br, I, and SCN) were prepared in high yields. All complexes possess linear geometry around the gold atom with no aurophilic interactions between neighboring molecules. Reactivity studies for (DAC)Au(I)Cl revealed that the diamido backbone of the carbene ligand is vulnerable to nucleophilic attack by a strong base, potassium *tert*-butoxide, resulting in cleavage of the carbene backbone and formation of a neutral trigold cluster. Halide and pseudohalide complexes are bright phosphorescent emitters in the solid state, exhibiting photoluminescence quantum yields up to unity. Phosphorescence occurs in the range 480–520 nm with lifetimes as short as 1 μs, resulting in fast radiative rates up to 9.4 × 10^5^ s^–1^ which is on par with the most efficient heavy metal emitters. Photophysical properties are explained by the intrinsic π-accepting nature of the DAC carbene and are supported by TDDFT calculations.

## Introduction

*N*-Heterocyclic carbenes (NHCs) are among the most successful and widely used ligands in organometallic chemistry.^1^ The diamidocarbene (DAC) is a unique subset of the NHC family, developed by Bielawski and co-workers, possessing increased π-accepting abilities due to the inclusion of carbonyl groups into the ligand backbone.^2^ The subsequent balance of nucleophilic and electrophilic character allows this carbene to perform transformations outside the remit of typical NHC’s. Exemplified in the works of Bertrand and Bielawski, DACs have been shown to undergo [2 + 1] cycloadditions with alkenes, aldehydes, alkynes, and nitriles. In addition, coupling to isocyanides results in the formation of *N*,*N*′-diamidoketeneimines.^3,4^ DACs have also been demonstrated to undergo reversible coupling to CO and mediate the dehydrogenation of hydrocarbons.^5^ The ability to perform these transformations can be attributed to the ambiphilicity of the DAC carbene.^3^ Five-, six-, and seven-membered DACs have been developed; however, a majority of the research has been concerned with the six-membered derivative owing to its facile synthesis and stability.^6^

NHC-carbene ligands paved the way toward bright photoluminescent organometallic complexes;^7^ the DAC carbene is no exception. Taking advantage of the DAC-carbene’s unique electronic properties, Whittlesey and Thompson reported various linear two-coordinate (DAC)Cu(I)Y complexes (Y = DAC, -OSiPh_3_, -C_6_F_5_, and 2,4,6-Me_3_C_6_H_2_, carbazoles) emitting sky blue to red light from 456 to 666 nm with photoluminescence quantum yields (PLQY) between 1 and 68% depending on the media and Y ligand.^8b^ The nature of the luminescence was ascribed to a phosphorescence or thermally activated delayed fluorescence (TADF, for Y = carbazole) mechanism depending on the ligand in trans-position to DAC carbene. More recently, Thompson et al. reported gold(I)aryl complexes with DAC and monoamidocarbene (MAC) carbenes.^9^ However, the performance of these (DAC)Au(I)Aryl (aryl = phenyl or 4-carbazolyl-phenyl) complexes were relatively poor with PLQY < 0.1%. It was explained that the lowest energy excitation of these complexes possessed metal-to-ligand charge transfer (MLCT) character, therefore excitation was accompanied by transient oxidation of the Au(I) center. This causes bending in the C_carbene_–Au–C_aryl_ moiety, resulting in rapid rates of nonradiative decay (Renner–Teller distortion).^10^ Suppression of the Renner–Teller distortion was achieved by increasing the electron donating ability of the aryl ligand to change the character of the lowest excited state from MLCT to intermolecular charge transfer (ICT). This was achieved with the (DAC)Au(4-diphenylamino-phenyl) complex, which showed an orange-red emission at 620 nm with PLQY up to 38% in polystyrene host via the TADF mechanism.^9^

While (DAC)M(I)Chlorides are synthetic precursors to the (DAC)M(amide) and (DAC)M(aryl) complexes, the remarkable photoluminescence behavior of the (DAC)Au(I)Halides has received very little attention. This oversight may be due to the general nonemissive behavior of (NHC)AuCl complexes Here, we report bright phosphorescent linear (DAC)Au(I)Halides and investigate the products of their reactions with strong nucleophilic bases.

## Results and Discussion

### Synthesis and Anion Exchange Reactions

**Scheme 1:**
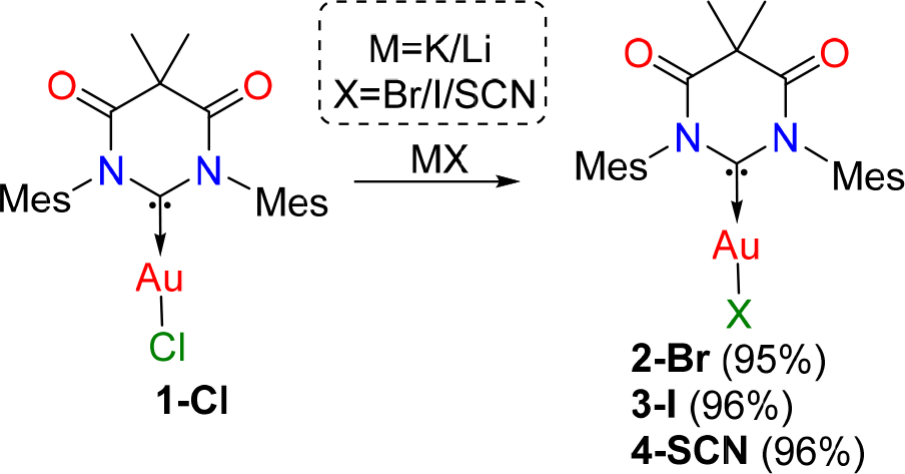
Synthesis of (DAC)AuX Complexes (X = Br, I, and SCN) from (DAC)AuCl via Anion Exchange Reactions

The chloride complex (DAC)AuCl (**1-Cl**) was obtained by reacting the carbene precursor DAC^mes^ClH with (Me_2_S)AuCl in the presence of potassium bis(trimethylsilyl)amide, following literature procedure.^11^ All other gold halide and pseudohalide complexes (**2-Br**, **3-I**, and **4-SCN**) were obtained in high yields via ligand substitution in acetone (**2-Br** and **3-I**) or ethanol (**4-SCN**, Scheme 1). All complexes are indefinitely stable in air. Characteristic ^13^C-carbene signals were observed in the ^13^C NMR spectra at 208, 210, 214 and 212 ppm for gold complexes **1-Cl** to **4-SCN**, respectively.

### Reactivity with KO^t^Bu Base

**Scheme 2:**
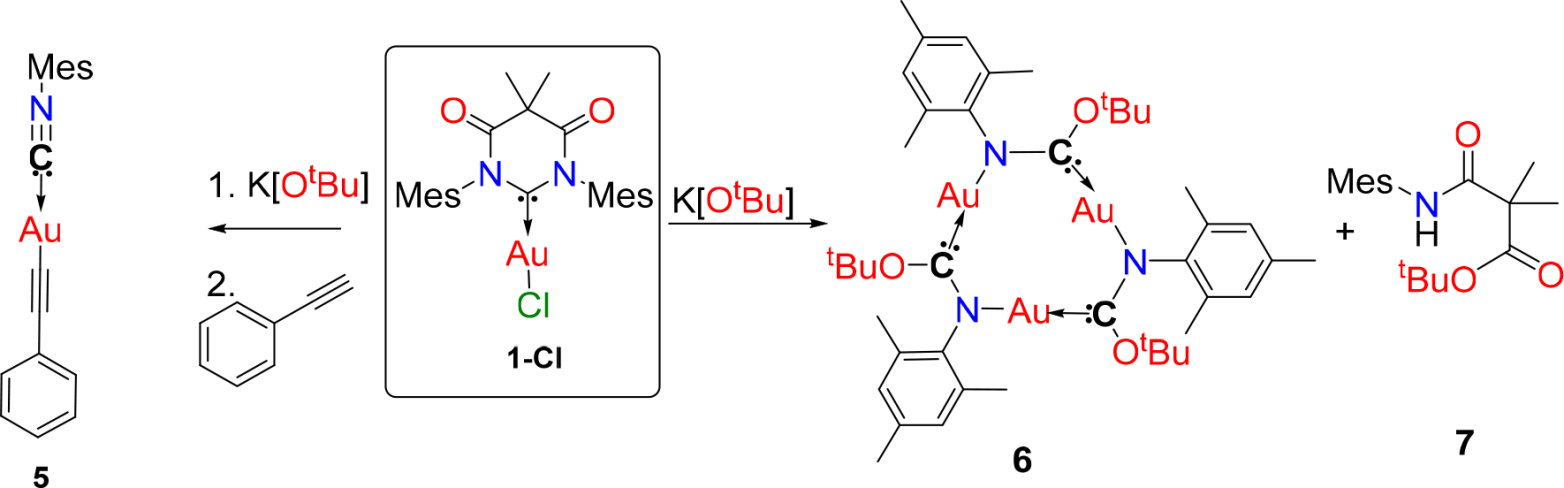
Synthesis of Isocyanide Complex **5** via Decapitation of the DAC Backbone and Gold Cluster **6** via Reaction of **1-Cl** with Potassium *tert*-Butoxide

**Scheme 3:**
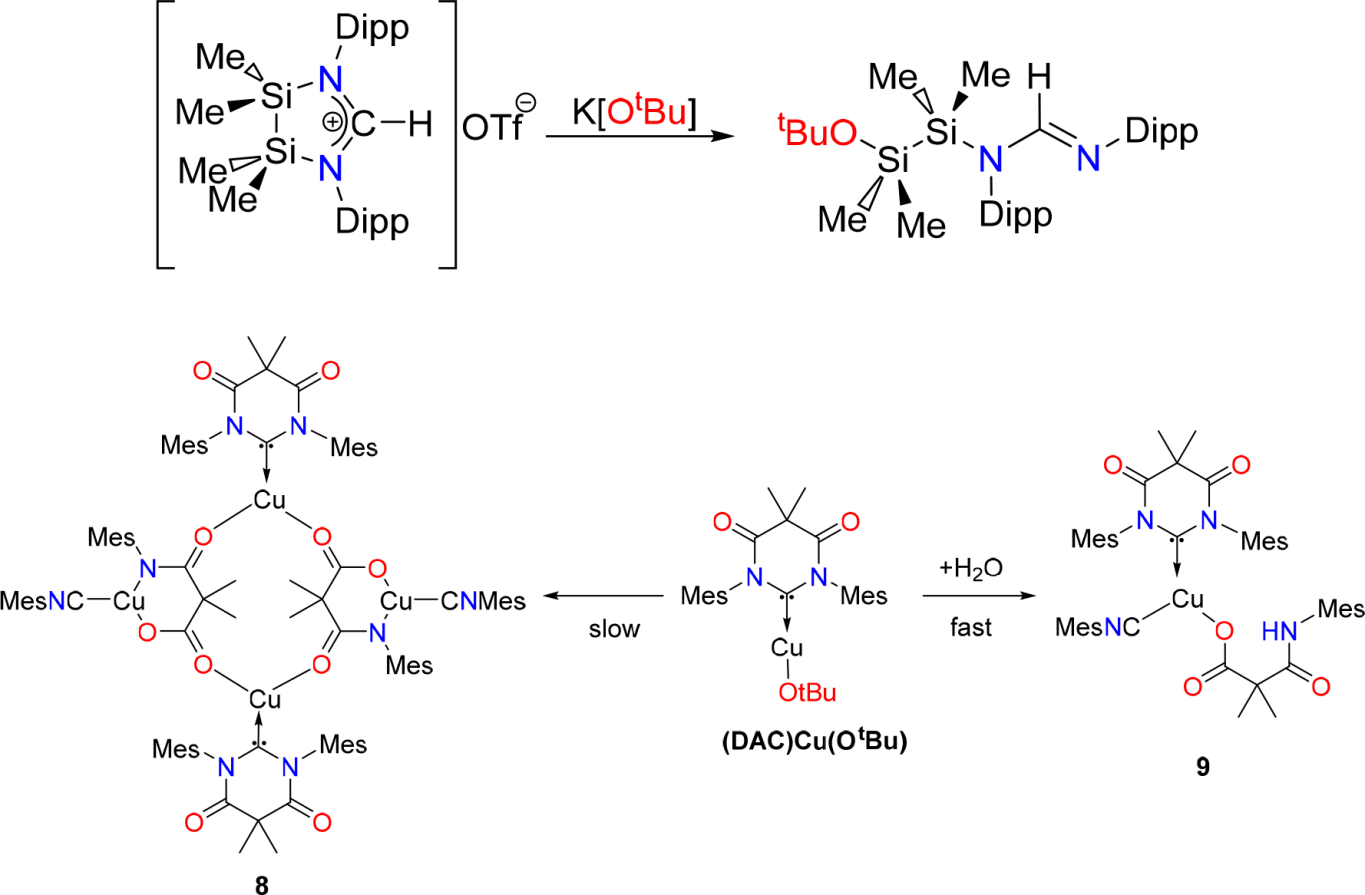
Nucleophilic Ring Opening of a Disilane Amidinium Salt by Reaction with KO^t^Bu (Top) and Degradation of (DAC)CuO^t^Bu as Demonstrated by Whittlesey et al. (Bottom)^8b^

During attempts to synthesize DAC-Au-Acetylides it emerged that the DAC carbene framework has a propensity to react with strong nucleophilic bases, such as KO^*t*^Bu. The reaction of **1-Cl** with phenyl acetylene in the presence of potassium *tert*-butoxide unexpectedly leads to the isolation of the isocyanide gold complex^12^**5** in 67% yield in place of the expected (DAC)Au(acetylide) as shown in Scheme 2. This intrinsic reactivity of **1-Cl** was further probed in the absence of the phenylacetylene by reacting **1-Cl** (1 equiv) and potassium *tert*-butoxide (2 equiv) to form the neutral trigold(I) cluster [Au(N^Mes^–CO^*t*^Bu)]_3_ (**6**, Scheme 2). Upon work up of the reaction mixture, hexane was used to wash the product **6**. Further examination of the hexane washings enabled us to isolate the remainder of the carbene backbone compound **7**. This suggests that cluster **6** is formed by nucleophilic attack at the C_carbene_–N bond by KO^t^Bu, resulting in ring opening of the DAC ligand. The structures of the compounds **5**–**7** are confirmed by NMR, high resolution mass spectrometry and X-ray diffraction studies (Scheme 3). The crystals of cluster **6** are relatively stable in air, while the solutions of **6** degrade rapidly in ambient light to form gold nanoparticles (deep purple solutions). A solid sample of **6** stored as a white powder at room temperature under N_2_ turned purple after one week, indicating the formation of the gold nanoparticles. The X-ray structure of fragment **7** is shown in Figure 2.

Nucleophilic degradation of NHC-carbene precursors, yielding ring opened products, has been previously documented.^13^ In pursuit of a NHC carbene containing a disilane backbone, Rivard et al. reported that deprotonation of a cyclic disilane amidinium salt [Me_2_NiNSipp)_2_CH]OTf resulted in nucleophilic NHC-ring opening (Scheme 3). Reactions with various bases, including KO^t^Bu, left the amidine N=*CH*–N moiety intact, while resulting in substituted formamidine in place of the desired NHC carbene (Scheme 3). Whittlesey et al. have previously demonstrated that the DAC ligand is susceptible to thermal and hydrolytic degradation.^8c^ As depicted in Scheme 3, the complex (DAC)Cu(O^t^Bu) degrades in solution to give a tetranuclear copper complex (**8**) or a mononuclear copper complex (**9**). The latter, complex **9**, contains organic fragments that strongly resembles compound **7**, isolated in this work. Both reported DAC degradation products **8** and **9** contain a mesityl nitrile ligand similar to complex **5** in this work.

**Chart 1:**
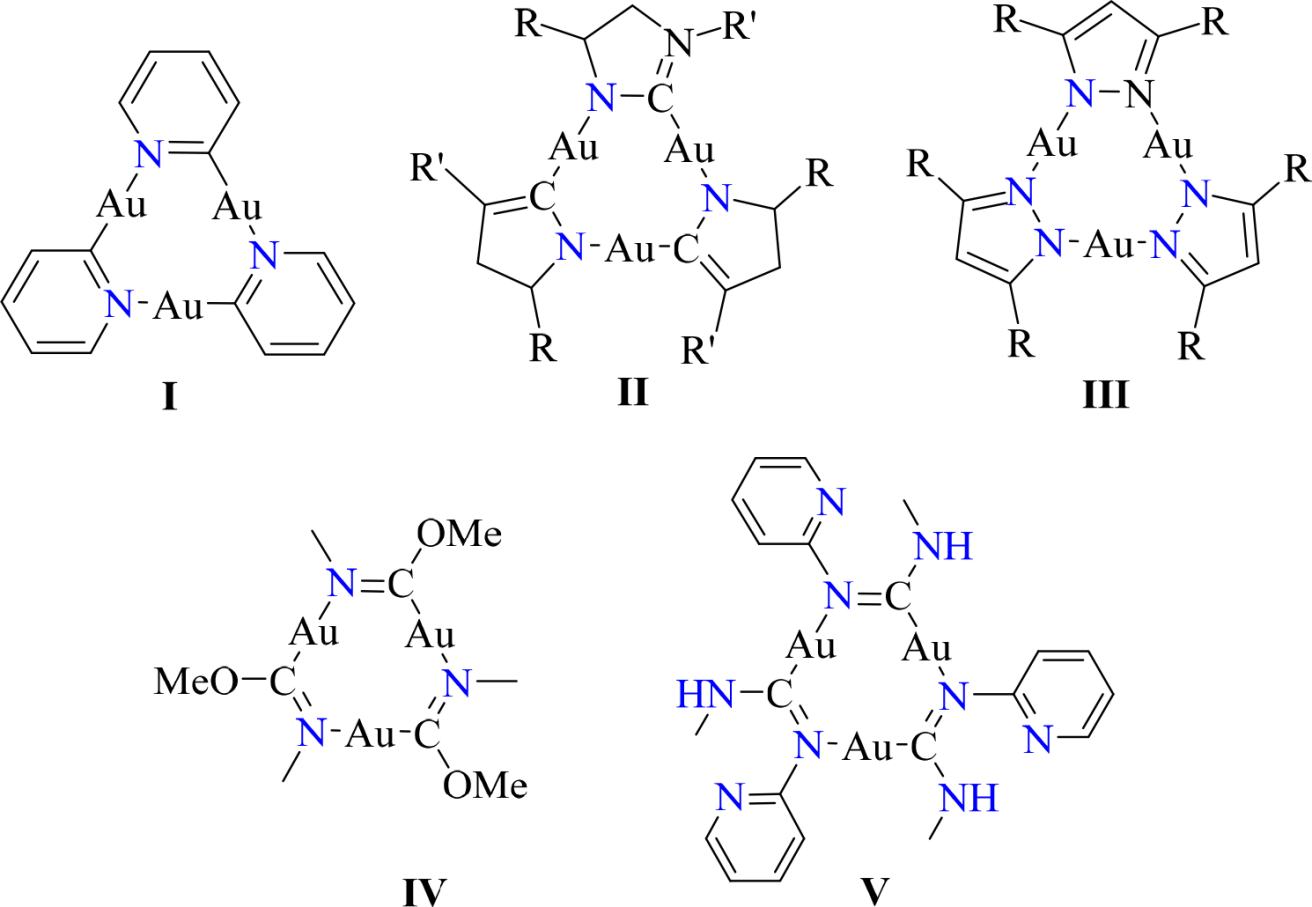


Similar to trigold cluster **6**, complexes of the type [AuL]_3_, where three Au(I) atoms are linearly coordinated by bridging ligands to form a planar nine-membered ring, have been known for over 50 years and are well explored in the works of Vaugh, Bonati, Fackler, and others.^13−16^ Notable examples of such structures are listed in Chart 1. Example **I** is one of the earliest known neutral Au(I) trimers, first proposed in 1969, when it was prepared by the addition of chloro(triphenylarsine)gold(I) to a solution of 2-pyridllithium.^13^ Imidazole containing gold clusters, such as example **II**, are prepared by first isolating the lithium salt of the substituted imidazole and subsequent reaction with PPh_3_AuCl in the presence of methanol.^14^ Example structures **III** and **IV** can be prepared by similar methods: A substituted pyrazole (**III**) or aromatic isocyanide (**IV**) is dissolved in methanol with a source of Au(I), typically Me_2_SAuCl or PPh_3_AuCl, in the presence of KOH.^15,17^ In this work, we achieved synthesis of the Au_3_ cluster by direct reaction of the carbene complex **1-Cl** with potassium *tert*-butoxide. This is a similar approach reported for the synthesis of trigold cluster **V**, where the reaction between carbene complex [AuCl(C(NHMe)(NHPy-2))] and KOH in methanol leads to the formation of **V** with a 32% yield.^17^ Our synthetic approach results in varied yields (26–63%) for the desired trigold cluster **6** due to its poor stability.

### X-ray Diffraction

**Figure 1:**
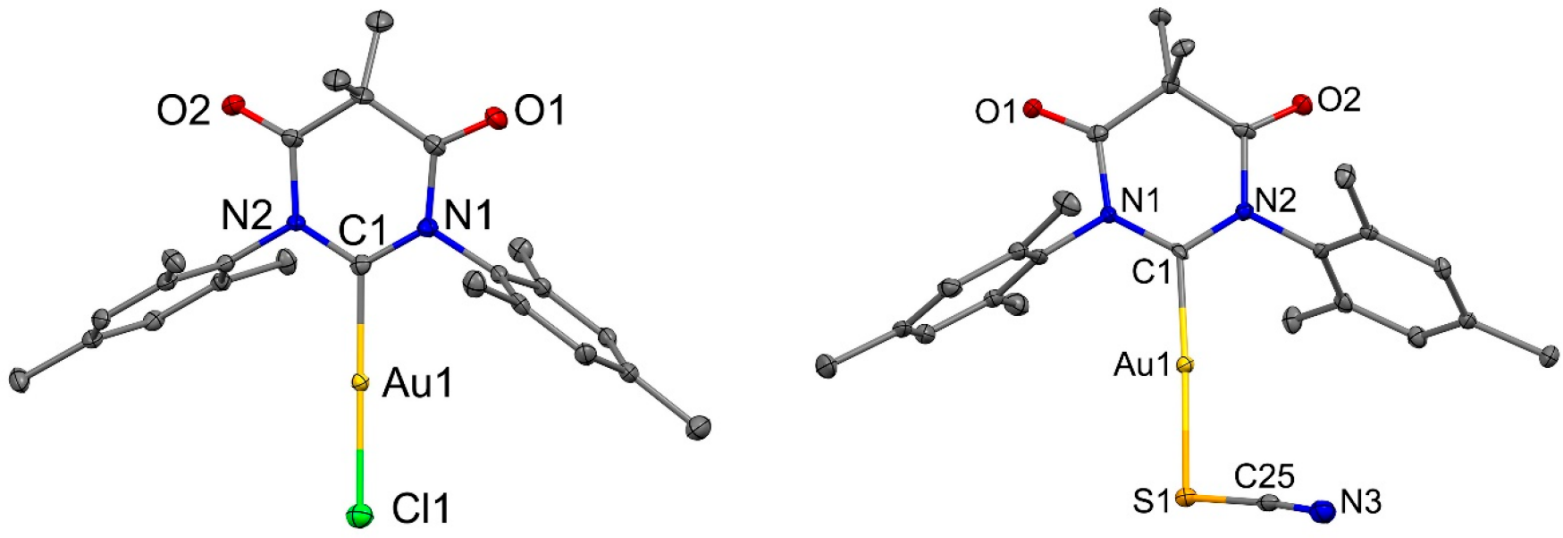
Crystal structure of chloride (DAC)AuCl (**1-Cl**, left) and thiocyanide (DAC)AuSCN (**4-SCN**, right). Ellipsoids are shown at the 50% level. Hydrogen atoms have been omitted for clarity.

Single crystals of all of the DAC-Au-X complexes suitable for X-ray diffraction studies were grown by layering a CH_2_Cl_2_ solution with hexane. The molecular structures are shown in Figure 1 for **1**-**Cl** (left)^6b^ and **4-SCN** (right) as representative examples, while key geometric parameters are collected in Table 1. All gold complexes crystallize with two independent molecules per unit cell, both having a two-coordinate linear geometry around the gold atom. Deviation from linearity in the C_1_–Au–X moiety is 3° larger for the pseudohalide **4-SCN** compared with gold halide complexes **1**–**3**. The Au–DAC carbene bond length experiences minor variations from 1.99 Å within the experimental error of 0.006 Å for all complexes. The elongation of the Au–X (X = Cl, Br, I, SCN) bond length follows the increase of the halide anion radii when descending the halide group, resulting in a larger separation between DAC-carbene and halide/pseudohalide atoms (Table 1). This separation impacts the molecular orbital overlap integral, which is directly proportional to the exchange energy between singlet and triplet excited states and radiative rates for the materials, *vide infra*. Analysis of intermolecular interactions indicated that none of the halide and pseudohalide gold complexes exhibit aurophilic (Au···Au) interactions. This is likely due to the bulky mesityl groups providing steric protection around the Au(I) atom. In Figure S19, where interlocking mesityl groups have been removed for clarity, it is possible to see the herringbone structure of the crystal packing. This is caused by weak intermolecular hydrogen bonds between the carbonyl groups and the CH_3_ groups of the mesityl moiety.

**Table 1:** Selected Bond Lengths (Å) and Angles (deg) for Gold Complexes **1**–**6**

	Au–C(1A), (Å)	Au–X, (Å)	C(1A)···X, (Å)	C(1A)–Au–X, (deg)
**1-Cl**^a^	1.993(5)	2.276(1)	4.264(5)	179.3(1)
**2-Br**^a^	1.989(4)	2.395(1)	4.370(4)	179.1(1)
**3-I**^a^	2.001(6)	2.541(1)	4.549(6)	178.4(1)
**4-SCN**^a^	2.010(5)	2.303(1)	4.313(5)	176.6(1)
**5**	1.973(11)	1.971(11)	3.950(5)	179.8(4)
**6 (Au3)**	1.987(5)	2.056(4)	4.044(5)	178.2(2)

a Averaged values for two independent molecules in the unit cell.

**Figure 2:**
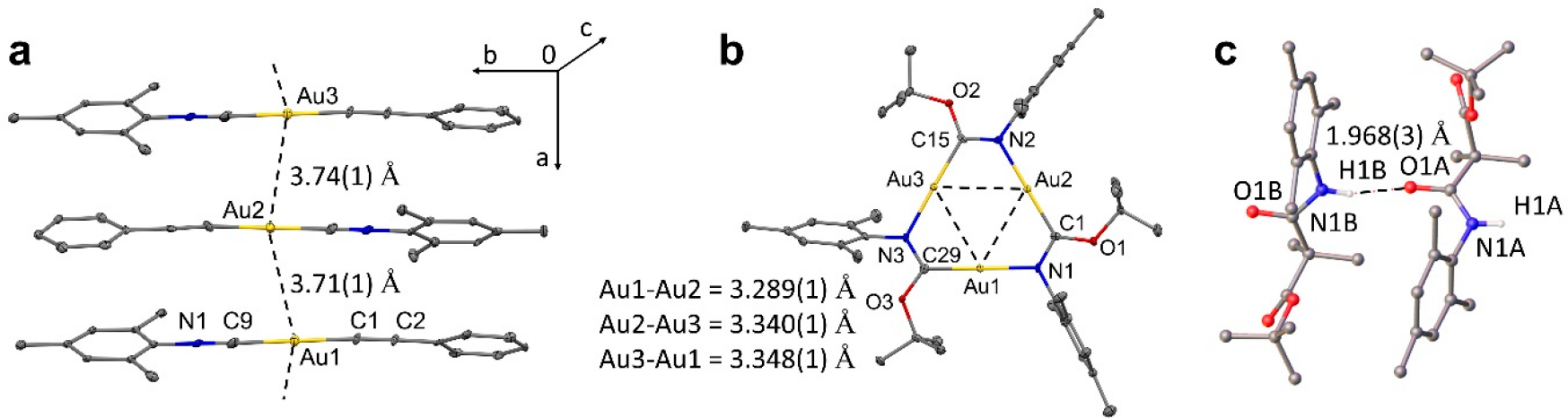
Molecular structures of complex **5** [(Au(C≡CPh)(CNC_6_H_2_Me_3_-2,4,6)] (a) showing the head-to-tail packing of **5** along crystallographic axis *a* due aurophilic interactions (dashed line); molecular structure of trigold cluster **6**; (b) the molecular structure of the organic fragment (**7**) found in washings of reaction shown in Scheme 2. Ellipsoids are shown at the 50% level. Hydrogen atoms are omitted for clarity except H1A and H1B for **7** showing a short hydrogen bond N1B–H1B···O1A between neighboring molecules.

The molecular structures of gold acetylide **5** and trigold cluster **6** complexes are shown in Figure 2. Both complexes possess a linear geometry around the gold atom. The Au–C (acetylide) and Au–C (isocyanide) bond lengths are 1.971(1) and 1.972(8) Å, both comparable to the previously reported complex [(Au(C≡CPh)(CNC_6_H_3_Me_2_-2,6)], which differs from **5** by a single methyl group.^12^ In the crystal lattice of [(Au(C≡CPh)(CNC_6_H_3_Me_2_-2,6)] two weakly interacting molecules pair up in an anti configuration. This head-to-tail packing style is also observed for complex **5** (Figure 2). However, the distance between gold atoms for **5** is 3.74(1) Å, which is at the upper limit for aurophilic interactions, and significantly larger than the 3.329(4) Å Au···Au distance in [(Au(C≡CPh)(CNC_6_H_3_Me_2_-2,6)].^14^ The increased intermolecular Au atom distances in **5** can be attributed to the torsional angle between the planes of the mesityl and phenyl groups, which is 37.5(1)°. This likely prevents the close packing of the complex in the solid state, elongating the aurophilic interactions. Unlike in complex **5**, the two phenyl rings in [(Au(C≡CPh)(CNC_6_H_3_Me_2_-2,6)] have a coplanar orientation.

Trigold cluster **6** possesses average C_carbene_–Au 1.987(5) Å and N_amide_–Au 2.056(4) Å bond distances similar to those measured for the mononuclear carbene-metal-amide materials.^18^ Typically the Au···Au distance for [AuL]_3_-type complexes is between 3.1–3.5 Å, for complex **6** the average Au–Au distance is of 3.325(1) Å which fits comfortably in this range. It is typical for [AuL]_3_ clusters to experience intermolecular aurophilic interactions that influence the supramolecular structure and crystal packing for such materials. A common motif is the formation of discrete dimers by vertical stacking of two [AuL]_3_ molecules having six gold atoms in corners of the trigonal prismatic array.^17^ Complex **6** has a structural motif similar to that of cluster **IV** shown in Chart 1. In cluster **IV** aggregation extends beyond simple dimer structures, the formation of columnar stacks is supported by extensive intermolecular aurophilic interactions which are responsible for the remarkable solvatochromic luminescence properties of **IV**. Furthermore the charge separation facilitated by the conduction of electrons through the columnar structures in {[AuL]_3_}_*n*_ oligomers hints at potential energy storage applications.^19^ However, for complex **6** the shortest intermolecular Au···Au distance is 6.3 Å (Figures 2 and S18), which is significantly larger than the 3.6 Å threshold value required to enable intermolecular aurophilic interactions. Such efficient separation between individual [AuL]_3_ molecules is due to the bulky mesityl groups, which are oriented perpendicular to the plane defined by the central nine-membered ring of complex **6**. Given that intermolecular aurophilic interactions usually originate the luminescence in {[AuL]_3_}_*n*_ oligomers, it is unsurprising that cluster **6** is nonemissive in the solid state due to absence of the intermolecular aurophilic interactions. DFT calculations of cluster **6** show a large orbital overlap integral of 0.65 (Table S3). The HOMO is unsymmetrically distributed across the cluster with relative contributions of 0.9, 12.5, and 22.6% from Au1, Au2, and Au3 atoms. In contrast, the LUMO for cluster **6** has equal contributions from each Au atom (16.9%), and the remaining electron density is dispersed evenly across the organic moiety of the trigold cluster.

### Electrochemistry

Cyclic voltammetry for halides (**1-Cl**, **2-Br**, **3-I**) and pseudohalide complex **4-SCN** was performed in 1,2-difluorobenzene (DFB, Figure S4) with data summarized in Table 2. All complexes show a reversible reduction process (Figure S4) with very small variation in *E*_1/2_ values at −1.60 ± 0.04 V, resulting in the similar values for the lowest unoccupied molecular orbital (LUMO) at −3.66 ± 0.04 eV. This suggests that LUMO is largely localized on the DAC ligand. Our previous work with cyclic(alkyl)(amino)Cu(I) and gold(I) halide complexes show irreversible reduction processes.^18,20^ Moreover, the energy of the LUMO for (CAAC)AuCl is 0.8 eV higher compared to the DAC-analogue (**1-Cl**) supporting the general trend that introduction of π-accepting carbonyl substituents in the carbene molecular structure stabilizes the LUMO energy level in the corresponding organometallic complex. The oxidation potential for all complexes is very close to the solvent discharge, preventing calculation of the energy for the highest occupied molecular orbital (HOMO). The HOMO energy level was estimated indirectly (*E*_*HOMO*_ = *E*_*opt-gap*_ – *E*_*LUMO*_) by measuring the optical energy gap (*E*_*opt-gap*_, eV) as the red onset of the lowest in energy absorption band in the UV–vis spectra in CH_2_Cl_2_ solution (see Table 2). The HOMO energy level strongly depends on the nature of the halide substituent. For instance, the HOMO is more stabilized for the chloride complex **1-Cl** (−7.62 eV) while it is destabilized for the iodide **3-I** (−6.72 eV), following the increase in the electronegativity of the halides (Cl > Br > I), thus predicting a red-shifted luminescence for the later.

**Table 2:** Formal Electrode Potentials (Peak Position *E*_*p*_ for Irreversible and *E*_*1/2*_ for Quasi-Reversible Processes (*), V vs. FeCp_2_), Onset Potentials (*E*, V vs. FeCp_2_), Peak to Peak Separation in Parentheses for Quasi-Reversible Processes (Δ*E*_*p*_, mV), and *E*_*LUMO*_ (eV) Complexes **1**–**4** and UV–vis in CH_2_Cl_2_ Solution^a^

	reduction					
compound	*E*_*first*_	*E*_*onset-red*_	*E*_*LUMO*_, eV	*E*_*HOMO*_, eV	*E*_*opt-gap*_, eV	λ_abs_ [nm], (10^3^ ε/M^–1^ cm^–1^)
**1-Cl**	–1.63(80)*	–1.74	–3.65	–7.62	3.97	262 (11), 283 (sh 5.8)
**2-Br**	–1.62(71)*	–1.73	–3.66	–7.34	3.68	262 (11), 287 (sh 5.7)
**3-I**	–1.64(86)*	–1.76	–3.63	–6.72	3.09	262 (13), 324 (3.8), 364 (3.7)
**4-SCN**	–1.55(90)*	–1.69	–3.70	–7.03	3.33	263 (13), 331 (6.6)

a In 1,2-difluorobenzene (DFB) solution, recorded using a glassy carbon electrode, concentration 1.4 m*M*, supporting electrolyte [^*n*^Bu_4_N][PF_6_] (0.13 *M*), measured at 0.1 V s^–1^. *E*_*LUMO*_ = – (*E*_*onset red Fc/Fc+*_ + 5.39) eV; *E*_*HOMO*_ = *E*_*opt-gap*_ – *E*_*LUMO*_; *E*_*opt-gap*_ defined as onset on the red edge of the lowest in energy absorption band in the UV–vis spectra.

### Photophysical Properties

**Figure 3:**
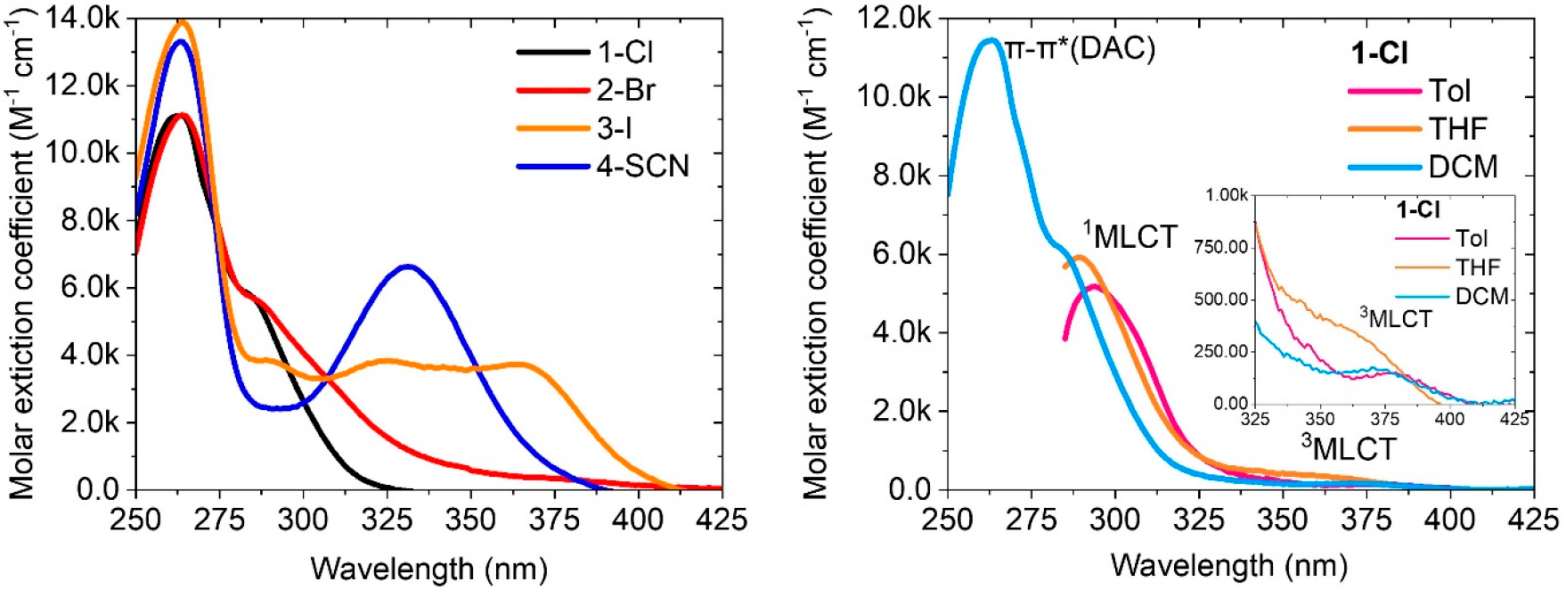
UV–vis spectra for all complexes in CH_2_Cl_2_ at 295K (left) and chloride complex **1-Cl** in various solvents (toluene, THF and CH_2_Cl_2_, right).

The UV–vis absorption spectra of the halide and pseudo halide complexes in CH_2_Cl_2_ solution are shown in Figures 3 and S5 and Table S2. All the complexes display a strong absorption at ca. 263 nm (ε> 11 × 10^3^ M^–1^cm^–1^) which has been assigned to an allowed intraligand π-π* transition on the DAC ligand (Figure 3 left panel). The UV–vis spectra for all complexes also contain a second, less intense absorption (ε ≈ 6 × 10^3^ M^–1^cm^–1^). Figures 3 and S5 show the UV–vis absorption spectra of each complex in CH_2_Cl_2_, THF and toluene solutions. For **1-Cl** and **2-Br** complexes, the secondary absorption displays ca. 10 nm blue shift upon increasing solvent polarity, indicating a charge transfer character. It has been assigned to a metal to ligand charge transfer ^1^MLCT transition supported by theoretical results (Table S3). Also, the **1-Cl** complex exhibits a very low intensity and broad absorption band (*ε* ≈ 300 M^–1^ cm^–1^) ascribed to a triplet ^3^MLCT transition which is supported by theoretical calculations (Tables S5 and S6). Complexes **3-I** and **4-SCN** show a more pronounced blue shift of 20 nm for the charge transfer absorption band (Figure S5) which is ascribed to the ^1^M(X)LCT transition (X = I and SCN).

**Figure 4:**
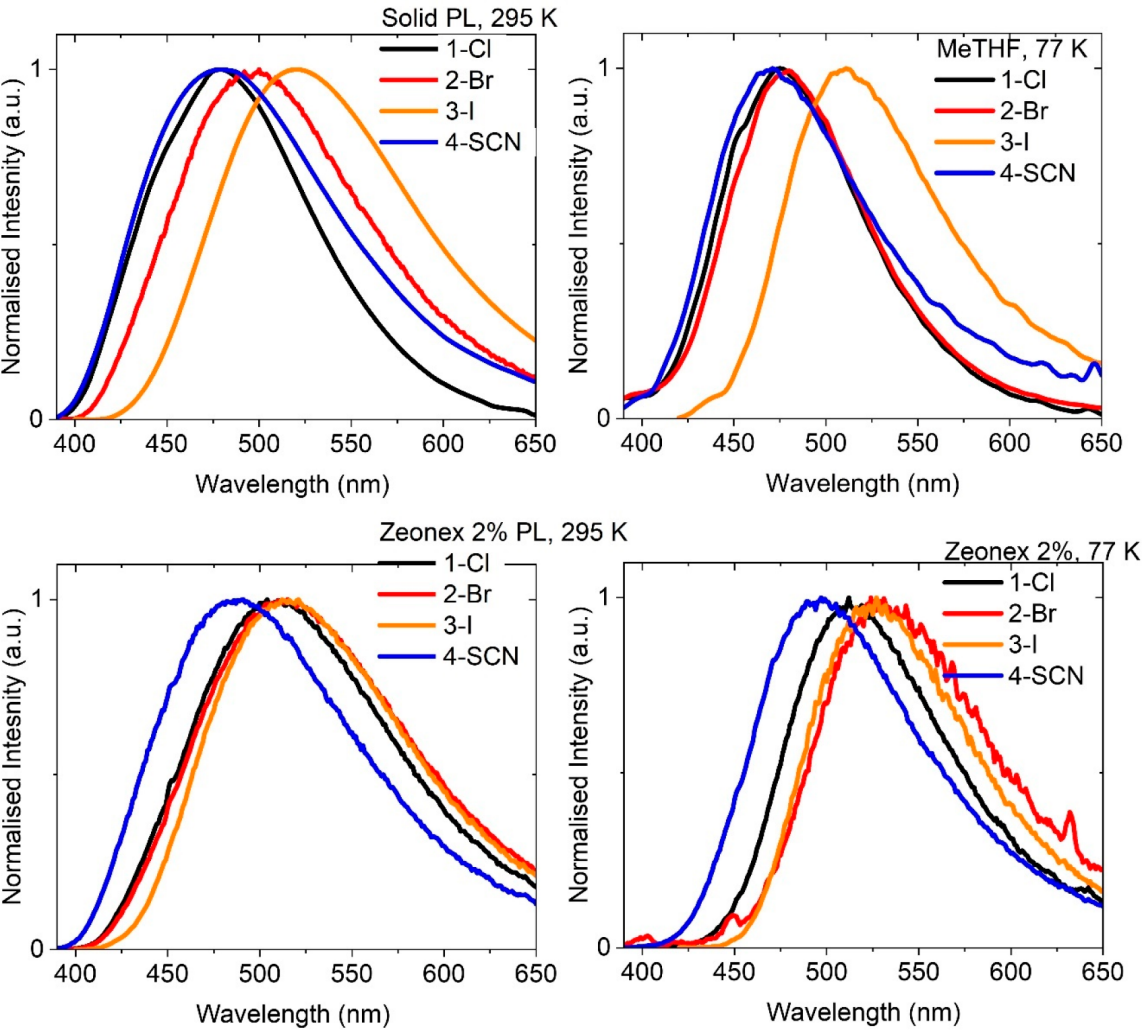
Emission spectra for (DAC)Au(X) complexes in the solid state at 295 K (top left); frozen MeTHF glasses at 77 K (top right); 2% by weight of the dopant in Zeonex at 298 K (bottom left) and 77 K (bottom right).

All halide and pseudohalide gold complexes show bright luminescence in the solid state as shown in Figure 4, while the photophysical data is summarized in Table 3. Complexes **1-Cl** and **4-SCN** show sky-blue emission, **2-Br** shows green emission, and **3-I** shows yellow-green emission. The emission profile is broad and featureless, indicative of an intramolecular charge transfer (ICT) state. Photoluminescence quantum yields (PLQY) are increasing from 86% for **1-Cl** to 97% for **3-I**, while **4-SCN** exhibits the lowest PLQY in the series up to 56%. All complexes in 2% Zeonex polymer films show bright phosphorescence for **4-SCN** (sky blue) and warm white for the halide complexes. The PLQY values in Zeonex follow the same trend and increase from 29% for **1-Cl** to 90% for **3-I**, while **4-SCN** show a minor decrease in PLQY down to 52% compared with the neat sample. Somewhat lower PLQY values for complexes in Zeonex films we associate with lower rigidity of the polymer media compared with the crystalline environment thus increasing the chances of the nonradiative events linked with the molecular geometry distortions. To the best of our knowledge, the near unity PLQY values exhibited by iodide complex **3-I** are the highest values reported to date across the luminescent carbene-metal-halides in both neat solid and polymer matrices.

**Table 3:** Photophysical Properies of the Gold Complexes **1-X** (X = Cl, Br, I, and SCN) in the Solid State and in Frozen MeTHF Glass, Overlap Integral between HOMO and LUMO Orbitals with Contribution of the Gold Atom

	λ_em_ (nm)	τ (μs)	Φ (%)^a^	*k*_r_ (10^5^ s^–1^)^b^	*k*_nr_ (10^5^ s^–1^)^c^	λ_em_ (nm)	τ (μs)	λ_em_ (nm)	*τ* (μs)	*T*_1_ (eV)	HOMO–LUMO overlap integral	Au contribution to HOMO (LUMO) (%)
Powder, 295 K						Powder, 77 K		MeTHF Glass, 77K				
**1-Cl**	480	2.4	86	3.6	0.58	476	2.8	477	3.5	3.00	0.23	16.7 (5.7)
**2-Br**	496	2.27	88	3.8	0.53	502	3.2	480	3.0	2.95	0.20	13.6 (5.8)
**3-I**	521	1.11	97	8.7	0.27	520	2.0	510	2.2	2.76	0.18	8.6 (5.9)
**4-SCN**	481	2.2	56	2.5	2.0	474	3.0	440	2.3	3.03	0.35	8.5 (5.5)
Zeonex 2% Film, 295 K						Zeonex 2% Film, 77 K						
**1-Cl**	504	2.34	29	1.2	3.0	511	4.1					
**2-Br**	515	1.89	56	2.9	2.3	527	3.27					
**3-I**	521	0.95	90	9.4	1.0	527	2.56					
**4-SCN**	487	2.3	52	2.2	2.1	495	3.49					

a Photoluminescence quantum yields (Φ) determined using an integrating sphere, excited at 320 nm.

b Radiative constant *k*_*r*_ = Φ/τ.

c Nonradiative constant *k*_*nr*_ = (1 – Φ)/τ.

All complexes possess phosphorescence excited state lifetimes (τ) in the range 0.95–2.4 μs (Table 3), which experience only marginal increase upon cooling to 77 K, indicating that phosphorescence is the luminescence mechanism for the title compounds. All complexes possess fast radiative rate constants (*k*_*r*_ = 1.2–9.4 × 10^5^ s^–1^) with the highest value 9.4 × 10^5^ s^–1^ for iodide complex **3-I**. Such fast radiative rates are comparable or superior to those reported for the most efficient phosphorescent octahedral Ir(III) or square planar Pt(II) (*k*_r_ ≈ 2–5 × 10^5^ s^–1^) complexes. Solutions of all complexes are very poorly emissive at room temperature. This is likely due to the increase in availability of nonradiative decay pathways (vibrational relaxation) in fluid media for complexes with linear geometry.^22^ Upon cooling to 77 K, all frozen solution MeTHF glasses exhibit bright phosphorescence (Figure 4) with up to a 40 nm blue shift compared to room temperature phosphorescence from the solid samples. The energy of the triplet state is estimated from the onset of the blue edge of the phosphorescence profile at 77 K in MeTHF (Table 3, Figure 4).

Previous theoretical works for linear carbene-M(I)-halides have found that bright phosphorescence requires the singlet S_1_ and triplet T_1_ wavefunctions have the same (X)MLCT character.^22,23^ This results in significant metal orbital contribution (30–50% to HOMO or LUMO) with large overlap between frontier orbitals stabilizing the triplet state energy compared to the singlet state, enabling bright phosphorescence in complexes with linear geometry.^20^ It was also noted that the presence of low-lying ligand based triplet states (^3^LC on aryl of the carbene ligand) does not promote intersystem crossing (ISC) or reverse ISC. The difference between the geometry of the ground and excited state needs to be considered to realize efficient phosphorescence from Carbene-M(I)-Halides. The series of copper complexes (CAAC)Cu(X) (X = Cl, Br, I)^21^ show phosphorescence in the solid state with PLQY up to 96%, whereas gold analogues (CAAC)Au(X) (X = Cl, Br, I) suffer from low PLQY values up to 18%. Theoretical calculation results explain that this due to significant deviation from linearity around metal center.

**Figure 5:**
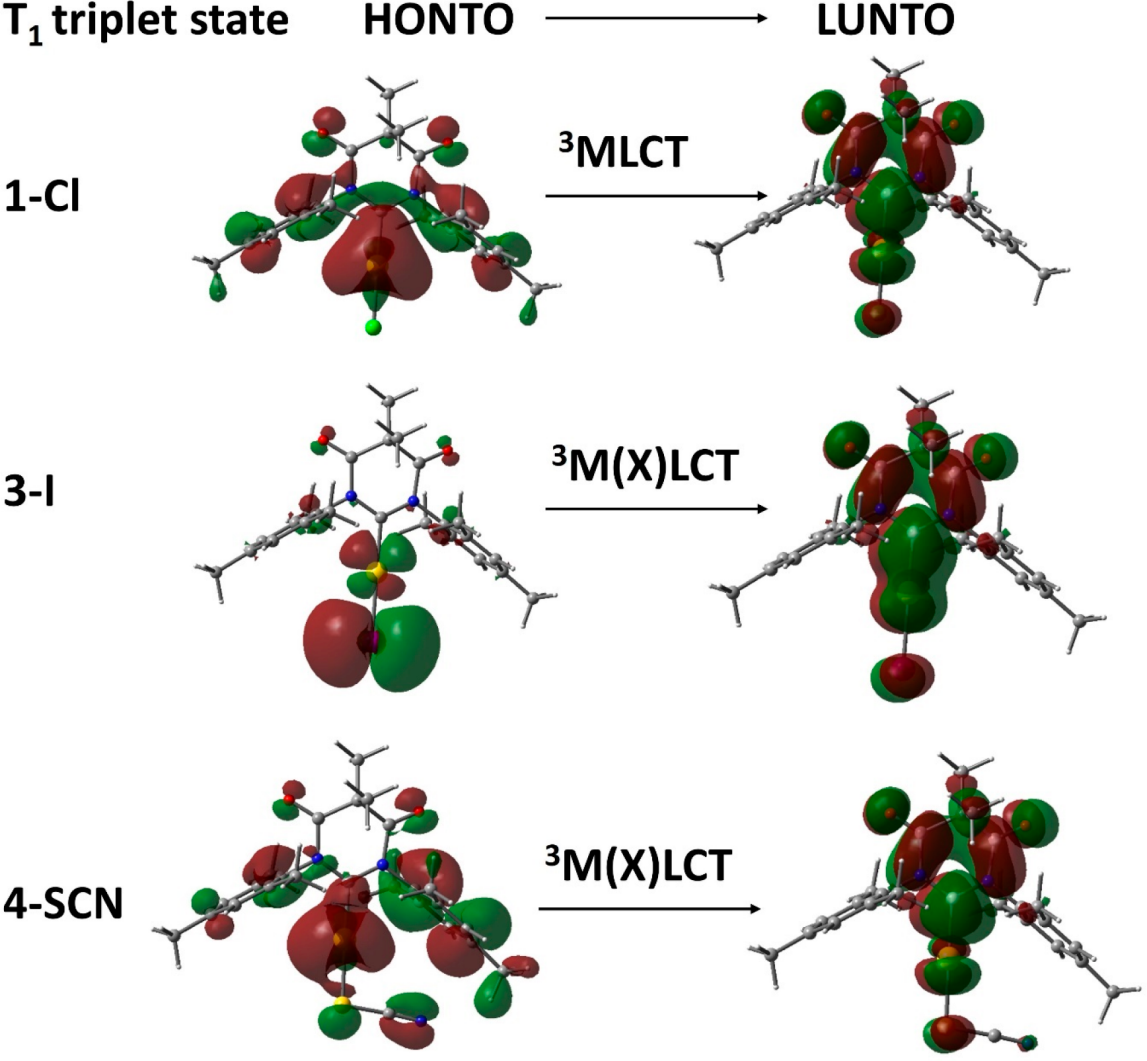
Highest occupied and lowest unoccupied NTOs (HONTO and LUNTO) for gold chloride (**1-Cl**), iodide (**3-I**), and pseudohalide (**4-SCN**) complexes.

DFT and TD-DFT calculations were performed to better understand the nature of the triplet excited state for phosphorescent gold complexes **1-Cl** to **4-SCN** containing the DAC-carbene ligand and compared with previous results. The isosurfaces for the calculated natural transition orbitals (NTOs) for **1-Cl** and **3-I** are shown in Figure 5 (Table S6). Theoretical calculations suggest only 6% gold contribution for the LUMO, which is largely localized over the DAC-carbene central core, but not on aromatic mesityl substituents. This is in marked difference to the classical NHC-carbene,^24^ and avoids having the low energy dark ^3^LC(aryl) state as a T_1_ state. The gold contribution is 16.7% for the HOMO of **1-Cl**, and gradually decreases for the remaining complexes (13.6% for **2-Br**, 8.6% for **3-I**, and 8.5% for **4-SCN**, Figure 5, Tables 3 and S3). Note that while the gold contribution to the HOMO decreases, the contribution of the halide or pseudohalide ligand gradually increases (Figure 5, Tables 3 and S3). Therefore, phosphorescence from **2-Br**, **3-I** and **4-SCN** complexes originates from the hybrid triplet ^3^M(X)LCT-state that involves a charge transfer from halide to DAC-carbene through the metal, whereas the chloride **1-Cl** complex exhibits the phosphorescence largely from the triplet ^3^MLCT state, see Figure 5 and Table S6.

Phosphorescence is a spin-forbidden process that involves a transition between states with different multiplicity. However, it is usually enhanced by the presence of an atom with a higher spin-obit coupling coefficient (H_SO_) due to relaxation of the spin selection rule, for instance, in (carbene)Au(I)halide complexes.^18^ The vital role of H_SO_ is further exemplified by increasing radiative rates for the series of gold halides in both solid state **1-Cl** (3.6 × 10^5^ s^–1^) < **2-Br** (3.8 × 10^5^ s^–1^) < **3-I** (8.7 × 10^5^ s^–1^) and Zeonex 2% films **1-Cl** (1.2 × 10^5^ s^–1^) < **2-Br** (2.9 × 10^5^ s^–1^) < **3-I** (9.4 × 10^5^ s^–1^). This trend parallels the increase of the halide atomic number (*Z*, where *H*_so_ ∝ *Z*^4^). This trend is also accompanied by a reduction in the overlap integral between HOMO and LUMO orbitals (Table 3) which decreases from chloride (0.23) to iodide (0.18) gold complexes. The fastest radiative rate of phosphorescence (9.4 × 10^5^ s^–1^) is observed for the gold iodide complex **3-I**, this is due to the small HOMO–LUMO overlap integral alongside the hybrid nature of triplet ^3^M(X)LCT-state. In contrast complex **4-SCN** displays the lowest phosphorescence radiative rate of 2.2 × 10^5^ s^–1^ which is likely associated with large HOMO–LUMO overlap integral of 0.35 and significant involvement of the DAC aryl ligand in the HONTO (Tables S3 and S6).

## Conclusion

A series of air and moisture stable DAC-carbene gold(I) halides (Cl, Br, and I) and thiocyanide complexes were synthesized by an anion exchange reaction in high yields. We demonstrated that a strong base, such as potassium *tert*-butoxide, reacts with the chloride complex (DAC)AuCl. This results in elimination of the DAC-carbene backbone and formation of either a neutral trigold cluster with structural type [AuL]_3_ or (isocyanide)gold(acetylide) complex in the presence of the phenylacetylene. Unlike many other linear carbene gold halide complexes that rely on aurophilic interactions to enable phosphorescence, we found that all title complexes show no aurophilic interactions while exhibiting bright phosphorescence with quantum yields up to 97% in both neat solids and 2% by weight doped films in Zeonex. Iodide complex **3-I** possesses the highest radiative rate of 9.4 × 10^5^ s^–1^ thanks to the heavy elements (Au and I) placing it on par with benchmark phosphorescent iridium(III) and platinum(II) organometallic compounds. Phosphorescence originates from either a triplet ^3^MLCT-state for chloride **1-Cl** or a hybrid triplet state ^3^M(X)LCT state for the bromide, iodide, and thiocyanide complexes. Theoretical results indicate that the choice of a strongly π-accepting carbene ligand avoids a dark ligand centered triplet ^3^LC state as the lowest in energy triplet state. This work suggests that future molecular design for fast and bright phosphorescent linear coinage metal complexes ought to focus on carbene ligands with a more π-accepting nature and conjugated backbone in comparison to conventional NHC-carbenes.

## Experimental Section

### General Considerations

All reactions were performed under a N_2_ atmosphere. Chemicals were purchased from commercial suppliers (Acros, Merck) and, unless otherwise noted, used as received. Solvents were dried as required. ^1^H and ^13^C were recorded by using a Bruker AVII HD 500 MHz spectrometer. ^1^H (500.19 MHz) and ^13^C (125.79 MHz) NMR spectra were referenced to CD_2_Cl_2_ at δ 5.32 (^13^C, δ 53.84) and CD_3_Cl at δ 7.26 (^13^C, δ 77.16). Mass spectrometry data was obtained by the Mass Spectrometry Laboratory at the University of Manchester. All electrochemical experiments were performed using an Autolab PGSTAT 302N computer-controlled potentiostat. Cyclic voltammetry (CV) was performed using a three-electrode configuration consisting of a glassy carbon macrodisk working electrode (GCE) (diameter of 3 mm; BASi, Indiana, U.S.A.) combined with a Pt wire counter electrode (99.99%; GoodFellow, Cambridge, U.K.) and an Ag wire pseudoreference electrode (99.99%; GoodFellow, Cambridge, U.K.). The GCE was polished between experiments using alumina slurry (0.3 μm), rinsed in distilled water, and subjected to brief sonication to remove any adhering alumina microparticles. The metal electrodes were then dried in an oven at 100 °C to remove residual traces of water, the GCE was left to air-dry and residual traces of water were removed under vacuum. The Ag wire pseudoreference electrodes were calibrated to the ferrocene/ferrocenium couple in 1,2-diflurobenzene (DFB) at the end of each run to allow for any drift in potential, following IUPAC recommendations. All electrochemical measurements were performed at ambient temperatures under an inert N_2_ atmosphere in the DFB containing the complex under study (0.14 mM) and the supporting electrolyte [*n*-Bu_4_N][PF_6_] (0.13 mM). Data were recorded with Autolab NOVA software (v. 1.11). Photoluminescence measurements were recorded using an Edinburgh Instruments FLS980 spectrometer. A xenon flashlamp was used as the excitation source. All excited state lifetimes were measured on FLS980 spectrometer with mono- and biexponential fitting provided by Edinburgh Instruments Fluoracle software v2.6.1. Solution UV–visible absorption spectra were recorded on a Cary 500 UV–vis-NIR spectrometer for a wavelength range 250–700 nm. Absolute photoluminescence quantum yields for the solid samples were recorded in air using Hamamatsu Quantaurus-QY C11347–11.

### X-ray Crystallography

Crystals were mounted in oil on a glass fiber and fixed on the diffractometer in a cold nitrogen stream. Data were collected using an Agilent SuperNova with Mo Kα (λ = 0.71073 Å) radiation at 100 K. Data were processed using the CrystAlisPro-CCD and -RED software.^25^ The structure was solved by intrinsic phasing or direct method and refined by the full-matrix least-squares against F2 in an anisotropic (for non-hydrogen atoms) approximation. All hydrogen atom positions were refined in isotropic approximation in a “riding” model with the *U*_iso_(*H*) parameters equal to 1.2 *U*_eq_(*C*_*i*_), for methyl groups equal to 1.5 *U*_eq_(*C*_*ii*_), where *U*(*C_i_*) and *U*(*C_ii_*) are, respectively, the equivalent thermal parameters of the carbon atoms to which the corresponding H atoms are bonded. All calculations were performed using the SHELXTL software.^26^ OLEX2 software was used as graphical user interface.^27^

### Computational Details

The ground states of the complexes were studied by density functional theory (DFT) and the excited states by time-dependent DFT (TD-DFT) using the Tamm–Dancoff approximation, as implemented in Gaussian 16.^28^ Calculations were carried out by the global hybrid MN15 functional^29^ of the Minnesota series in combination with the def2-TZVP basis set,^30^ employing the relativistic effective core potential of 60 and 28 electrons for the description of the core electrons of Au and I, respectively.^31^ This methodology has been employed in several papers dealing with closely related complexes, in good agreement with experiments.^32^ Gold contributions to HOMO and LUMO orbitals were evaluated by Mulliken population analysis. HOMO–LUMO overlap integrals were calculated using Multiwfn program.^33^

### Synthesis of DACAuCl (**1-Cl**)

A Schlenk flask charged with DAC^Mes^ClH (500 mg, 1.21 mmol, 1 equiv), KHMDS (254 mg, 1.27 mmol 1.05 equiv), and (Me_2_S)AuCl (339 mg, 1.15 mmol, 0.95 equiv) was cooled to −78 °C and dry THF (15 mL) added. The reaction mixture was then allowed to warm to room temperature and stirred for 4 h. The solution was then filtered through 4 cm of silica and eluted with more THF. Solvent was removed under reduced pressure to give the product as a white solid (501 mg, 68%).

^1^H NMR (500 MHz, CDCl_3_): δ 7.01 (s, 4H, H_a_) 2.23(s, 6H, H_b_) 2.16(s, 12H, H_c_) 1.80(s, 6H, H_d_). ^13^C(^1^H) NMR (75 MHz, CDCl_3_): δ 208.0 (C:, C_1_) 171.3 (C=O, C_2_) 140.5 (C, C_5_) 135.3 (C, C_8_) 134.1 (C, C_6_) 130.3 (CH, C_7_) 51.52 (C, C_3_) 24.9 (C_4_) 21.37 (CH_3_, C_9_) 18.2 (CH_4_, C_10_)

HRMS C_24_H_28_AuClN_2_O_2_ theoretical 608.1499; APCI 608.1497

### Synthesis of DACAuBr (**2-Br**)

Acetone (10 mL) was added to a flask charged with **1-Cl** (120 mg, 0.2 mmol, 1 equiv) and LiBr (174 mg, 2.0 mmol, 10 equiv) and stirred overnight at room temperature. Solvent was removed under reduced pressure, and the white solid was extracted into CH_2_Cl_2_ (15 mL) and filtered through celite. The volume of solvent was reduced, and the product precipitated by addition of hexane, centrifuged, and decanted yielding a white solid (125 mg, 95%).

^1^H NMR (500 MHz, CDCl_3_): δ 7.01 (s, 4H, H_a_) 2.34(s, 6H, H_b_) 2.16(s, 12H, H_c_) 1.81(s, 6H, H_d_). ^13^C(^1^H) NMR (75 MHz, CDCl_3_) δ 210.1 (C:, C_1_) 171.4 (C=O, C_2_) 140.5 (C, C_5_) 135.2 (C, C_8_) 134.1 (C, C_6_) 130.2 (CH, C_7_) 51.5 (C, C_3_) 24.9 (C_4_) 21.4(CH_3_, C_9_) 18.1 (CH_4_, C_10_)

HRMS [C_24_H_28_AuBrN_2_O_2_]^+^ theoretical 652.0994; APCI 652.1000.

### Synthesis of DACAuI (**3-I**)

A flask charged with **1-Cl** (120 mg,0.2 mmol, 1 equiv) and KI (332 mg, 2.0 mmol, 10 equiv) in acetone (10 mL) was stirred overnight at room temperature. Solvent was removed under reduced pressure and the white solid extracted into CH_2_Cl_2_ (15 mL) and filtered through celite. The volume of solvent was reduced, and the product precipitated by addition of hexane, centrifuged, and decanted yielding a white solid (125 mg, 96%).

^1^H NMR (500 MHz, CDCl_3_): δ 7.01 (s, 4H, H_a_) 2.34(s, 6H, H_b_) 2.16(s, 12H, H_c_) 1.81(s, 6H, H_d_). ^13^C(^1^H) NMR (75 MHz, CDCl_3_): δ 214.4 (C, C_1_) 171.6 (C=O, C_2_) 140.5 (C, C_5_) 135.0 (C, C_8_) 134.2 (C, C_6_) 130.1 (CH, C_7_) 51.6 (C, C_3_) 24.9 (C_4_) 21.37 (CH_3_, C_9_) 18.1 (CH_4_, C_10_)

HRMS [C_24_H_28_AuIN_2_O_2_] + H theoretical 701.0934; APCI 701.0949.

### Synthesis of DACAuSCN (**4-SCN**)

A flask charged with **1-Cl** (120 mg, 0.2 mmol, 1 equiv) and KSCN (192 mg, 2.0 mmol, 10 equiv) in ethanol (10 mL) was stirred overnight at room temperature. Solvent was removed under reduced pressure and the white solid extracted into CH_2_Cl_2_ (15 mL) and filtered through celite. The volume of solvent was reduced, and the product precipitated by addition of hexane, centrifuged, and decanted yielding a white solid (120 mg, 96%).

^1^H NMR (500 MHz, CDCl_3_): δ 7.04 (s, 4H, H_a_) 2.34(s, 6H, H_b_) 2.18 (s, 12H, H_c_) 1.83 (s, 6H, H_d_). ^13^C(^1^H) NMR (75 MHz, CDCl_3_) δ 211.8 (C, C_1_) 171.4 (C=O, C_2_) 141.2 (C, C_5_) 134.6 (C, C_8_) 135.0 (C, C_6_) 130.44 (CH, C_7_) 115.1 (SCN, C_11_) 51.7 (C, C_3_) 25.0 (C_4_) 21.4 (CH_3_, C_9_) 18.2 (CH_4_, C_10_). HRMS C_25_H_29_AuSN_3_O_2_ theoretical 632.1649; APCI 632.1641.

### Synthesis of **5** (MesC≡N)AuC≡CPh

A 100 mg portion of **1-Cl** (0.16 mmol) and 20 mg of potassium *tert*-butoxide (0.18 mmol) were dissolved in dry THF and cooled to −78 °C. Phenyl acetylene (17 mg, 0.16 mmol) was added dropwise, and the mixture was allowed to warm to room temperature and stirred overnight. All volatiles were removed under vacuum and the product precipitated by the addition of dry pentane. Yield: 47 mg, 67%.

^1^H NMR (500 MHz, CD_2_Cl_2_): δ 7.46 (d, 2H, Ar *J* = 7.17 Hz) 7.25–7.18 (m, 3H) 6.96 (s, 2H, Mes H) 2.38 (s, 6H, Mes) 2.32 (s, 3H, Mes). ^13^C(^1^H) NMR (75 MHz, CDCl_3_): δ 141.8; 136.1; 132.6; 129.3; 128.1; 127.1; 124.7; 122.0; 104.1 (CC); 21.5 (CH_3_); 18,7 (CH_3_).

HRMS [C_18_H_16_AuN] + H theoretical 444.1021; APCI 444.1021.

### Synthesis of Au_3_ Cluster **6**

130 mg of **1-Cl** (0.21 mmol) and 72 mg of potassium *tert*-butoxide (0.64 mmol) were cooled to −78 °C before the slow addition of dry THF (15 mL). The reaction was left to warm to room temperature and stirred overnight. Solvent was removed under reduced pressure, and the product extracted into dry CH_2_Cl_2_, filtered through celite. Removal of solvent gave the product was a white powder which was washed with pentane. Yield: 23–56 mg (26–63%).

^1^H NMR (500 MHz, CD_2_Cl_2_): δ 6.89 (6H, Ar) 2.27 (30H, alkyl), 1.50 (12H, alkyl), 1.14 (9H, alkyl). Due to instability of the cluster in solution, acquisition of ^13^C NMR of acceptable quality was not possible. HRMS C_42_H_61_Au_3_N_3_O_3_ theoretical 1246.3714; APCI 1246.3704.

## Supporting Information Available

^1^H and ^13^C{^1^H} NMR spectra, electrochemistry, HRMS, photoluminescence, TDDFT data for all compounds (PDF)
Coordinates for all computational structures (XYZ)

## Supplementary Material

om3c00360_si_001.pdf

om3c00360_si_002.xyz
