# Supplementary material for: Linear Gold(I) Halide Complexes with a Diamidocarbene Ligand: Synthesis, Reactivity, and Phosphorescence
Source: Organometallics. 2023 Nov 20;43(16):1687–97. doi: 10.1021/acs.organomet.3c00360 (PMC11351423; doi:10.1021/acs.organomet.3c00360)
Supplement: Supplementary file 1 — om3c00360_si_001.pdf [file om3c00360_si_001.pdf]

Supporting Information For:

Linear Gold(I) Halide Complexes with a Diamidocarbene Ligand: Synthesis, Reactivity and Phosphorescence

Charlotte Riley, [1] Nguyen Le Phuoc, [2] Mikko Linnolahti, [2*] Alexander S. Romanov [1*]

[1]Department of Chemistry, The University of Manchester, Oxford Rd. Manchester, M13 9PL, United Kingdom

[2]Department of Chemistry University of Eastern Finland, FI-80101 Joensuu, Finland

Corresponding Author:

*E-mail: alexander.romanov@manchester.ac.uk

*E-mail: mikko.linnolahti@uef.fi

Crystallographic data	p. S2
Electrochemistry	p. S7
Photophysical Measurements	p. S8
NMR spectra of complexes	p. S11
Mass spectra of complexes	p. S17
Computational details	p. S20

Table S1. Summary of crystallographic data and structure refinement

	**1-Cl** DAC-Au-Cl	**2-Br** DAC-Au-Br	**3-I** DAC-Au-I	**4-SCN** DAC-Au-SCN	**5** MesC≡NAuC≡CPh
CCDC №	2287802	2287803	2287804	2287805	2287806
Empirical formula	C24H28AuClN2O2	C24H28AuBrN2O2	C24H28AuIN2O2	C25H28AuSN3O2	C18H16AuN
Molecular weight	608.90	653.36	700.35		443.23
Crystal system	Monoclinic	Monoclinic	Monoclinic	Monoclinic	Monoclinic
Space group	P21/c	P21/c	P21/c	P21/c	P21/c
Crystal colour, habit	colourless block	colourless block	colourless block	colourless block	colourless block
Crystal size (mm)	0.39 ×0.37×0.22	0.39 ×0.35×0.14	0.39 ×0.29×0.23	0.29 ×0.22×0.16	0.42 ×0.23×0.15
a (Å)	11.7362(3)	11.8430(4)	12.0302(2)	12.0800(3)	7.2537(2)
b (Å)	20.3387(4)	20.3460(6)	20.5406(3)	20.3165(5)	45.3548(16)
c (Å)	20.3546(4)	20.2928(6)	20.2653(4)	20.2295(3)	13.7264(5)
β (°)	98.819(2)	98.831(3)	98.9284(16)	98.040(3)°	101.069(4)
V ($Å^3$)	4801.22(18)	47317(2)	4947.00(14)	4916.0(2)	4431.8(3)
Z	8	8	8	8	12
Dcalc (g cm^{-3})	1.685	1.796	1.881	1.881	1.993
2θmax (°)	53.998	58.196	51.998	51.998	56
Abs. coeff., (Mo-K) (mm^{-1})	6.261	7.7665	7.1073	6.096	9.947
T (K)	100.01(10)	100.01(10)	100.01(10)	100.01(10)	100.01(10)
Number of collected reflections	40226	22651	41129	41129	21082
Number of independent reflections	10378	11159	9682	9682	10055
R_{int}	0.0513	0.0387	0.0637	0.0637	0.0820
Number of parameters	559	557	559	593	406
R_1 (on F for observed reflexions)	0.0366	0.0374	0.0449	0.0647	0.0577
wR_2 (on F2 for all reflexions)	0.0881	0.0600	0.0806	0.0708	0.1195
F(000)	2384.0	2528.0	2672.0	2480.0	2520
Goodness-of-fit	1.028	1.003	1.054	1.022	1.024

	6<br>$Au_3(N^{mes}–CO^tBu)_3$	7<br>Backbone
CCDC №	2287807	2287808
Empirical formula	$C_{42}H_{60}Au_3N_3O_3$	$C_{18}H_{27}NO_3$
Molecular weight	1245.83	305.40
Crystal system	monoclinic	triclinic
Space group	P21/n	P-1
Crystal colour, habit	colourless block	colouless block
Crystal size (mm)	0.29 ×0.25×0.1	0.26×0.21×0.02
a (Å)	14.6420.(4)	9.6779(12)
b (Å)	13.9028(4)	17.531(2)
c (Å)	22.2736(6)	23.218(3)
α (°)	90	71.413(12)
β (°)	91.396(3)	81.372(11)
γ (°)	90	75.924(11)
V ($Å^3$)	4532.8(2)	3610.3(9)
Z	4	8
Dcalc (g cm^{-3})	1.826	1.124
2θmax (°)	53.998	52
Abs. coeff., (Mo-K) (mm^{-1})	9.723	0.076
T (K)	100.00(10)	100.01(10)
Number of collected reflections	21772	28693
Number of independent reflections	9719	14134
R_{int}	0.0430	0.1329
Number of parameters	478	825
R_1 (on F for observed reflexions)	0.0512	0.3267
wR_2 (on F2 for all reflexions)	0.0628	0.4939
F(000)	2376.0	1328.0
Goodness-of-fit	1.012	1.138

The A level alert for CCDC 2287808 is due to systematic twinning problem (regardless of several attempts to regrow the crystals of organic compound **7**) and very small size of the crystal. The quality of the structure was only sufficient to establish the connectivity between the atoms in isotropic approximation.

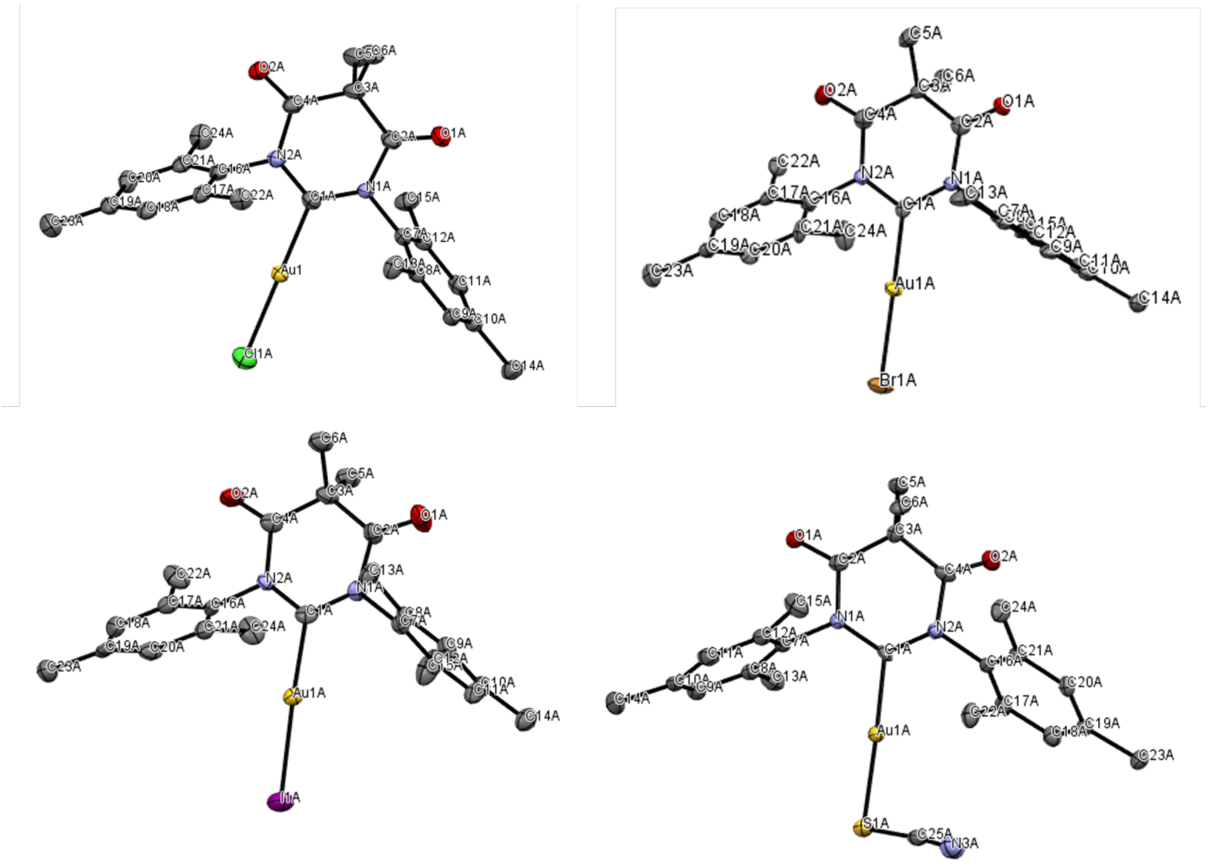

Figure S1. X-Ray crystal structures of DAC-Au-X complexes. Ellipsoids are shown at the 50% level. Hydrogen atoms emitted for clarity.

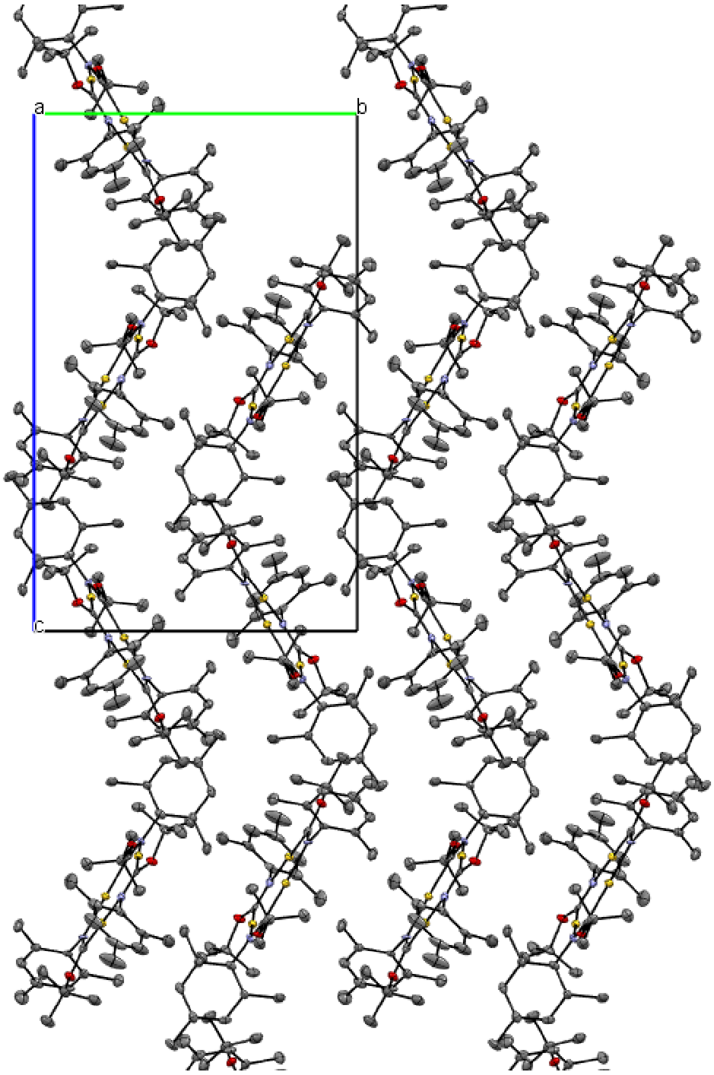

Figure S2. Crystal packing of cluster **7** in the solid state. View along ‘a’ axis of the unit cell. Hydrogen atoms are emitted for clarity.

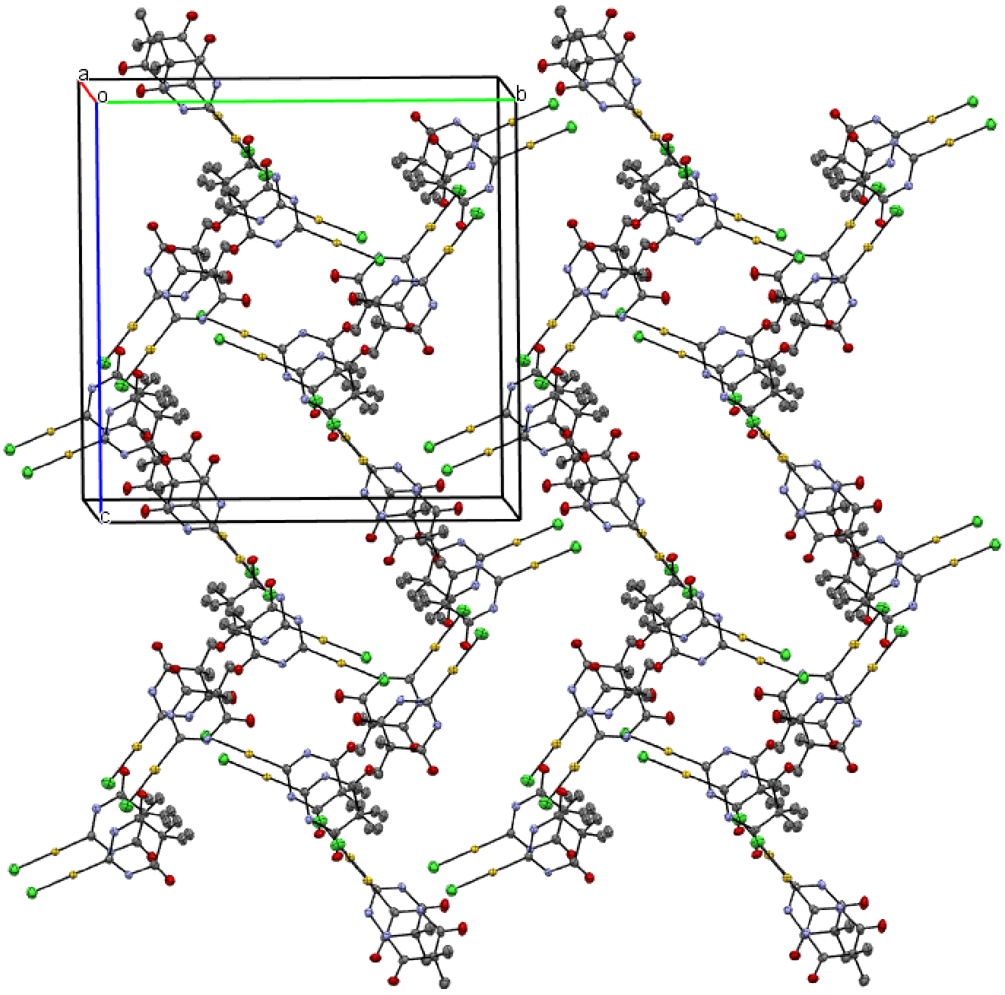

Figure 3. Crystal packing of complex DAC-Au-Cl (**1-Cl**). Interlocking mesityl groups and hydrogen atoms have been omitted for clarity.

Electrochemistry.

Electrochemical analysis was made for the complexes **DAC^{mes}AuX** (**X** = Cl/Br/I/SCN/OTf) using a glassy carbon electrode in DFB solution (1.4 mM) with [n-Bu$_4$]PF$_6$ as supporting electrolyte (0.13 M), scan rate 0.1 Vs^{-1}

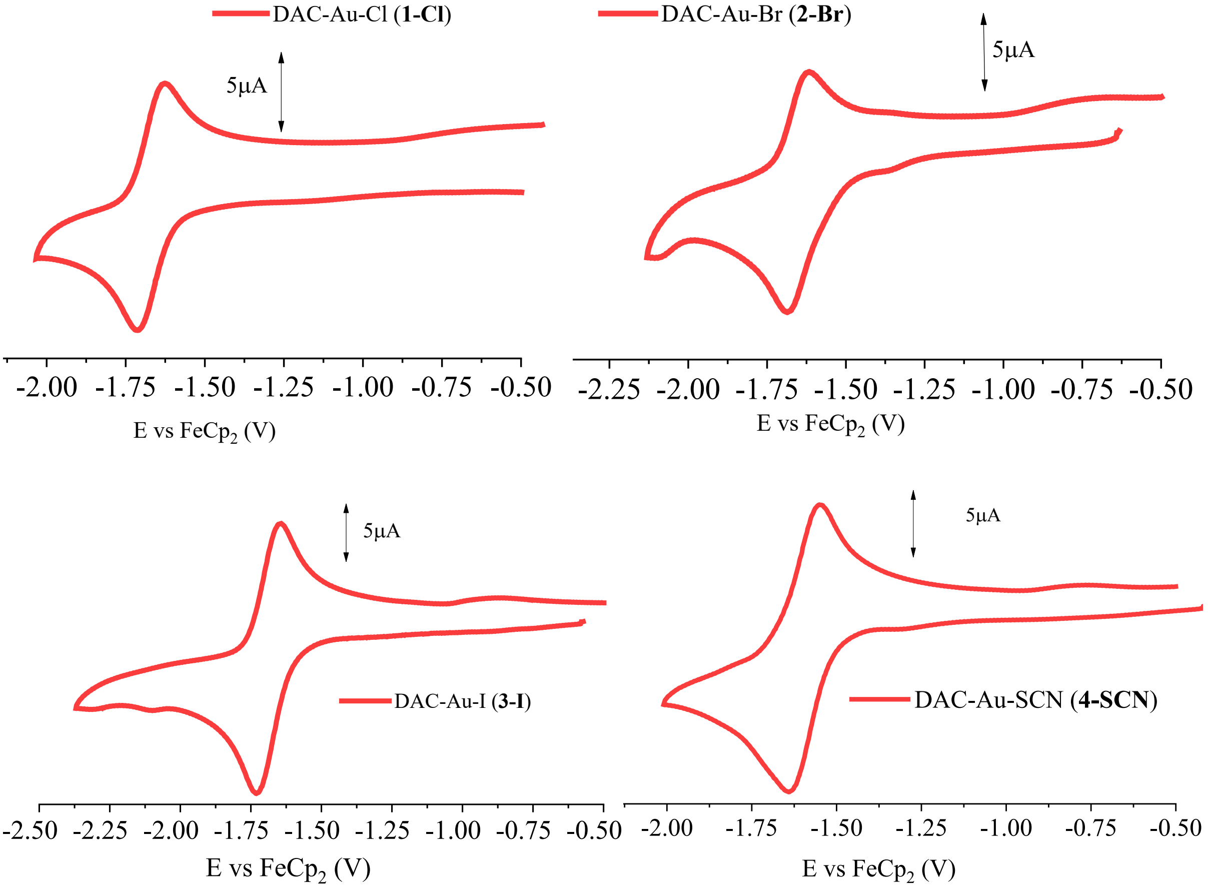

Figure S4. Cyclic voltammogram for reduction process exhibited by the complexes **1-Cl** (top left), **2-Br** (top right), **3-I** (bottom left), **4-SCN** (bottom right). Recorded using a glassy carbon electrode in 1,2-difluorobenzene (DFB) solution with concentration for the gold complex 1.4 mM and [n-Bu$_4$]PF$_6$ as supporting electrolyte (0.13 M), scan rate 0.1 Vs^{-1}.

Photophysical characterisation

Photoluminescence measurements were recorded using an Edinburgh Instruments FLS920 spectrometer. Absolute photoluminescent quantum yields were measured directly with a Quantaurus-QY Absolute PL quantum yield spectrometer. All excited state lifetimes were measured on FLS920 spectrometer with mono- and biexponential fitting provided by Edinburgh Instruments Fluoracle software v2.6.1.Solution UV-visible absorption spectra were recorded on a Cary 500 UV-vis-NIR spectrometer for a wavelength range 250-700 nm.

Table S2. UV-vis data for complexes **DAC^{mes}AuX** (**X** = Cl, Br, I or SCN) in CH_2Cl_2, THF and Toluene solution

Compounds	λ_{abs} [nm], (10^3 ε/M^{-1} cm^{-1})		
	CH_2Cl_2	THF	Toluene
**1-Cl**	262 (11) 283 (sh 5.8)	289 (5.9)	294 (4.8)
**2-Br**	262 (11) 287 (sh 5.7)	300 (4.2)	309 (3.0)
**3-I**	262 (13) 324 (3.8) 364 (3.7)	324 (4.2)370 (4.5)	338 (7.3) 382 (6.9)
**4-SCN**	263 (13) 331 (6.6)	337 (8.5)	350 (8.5)

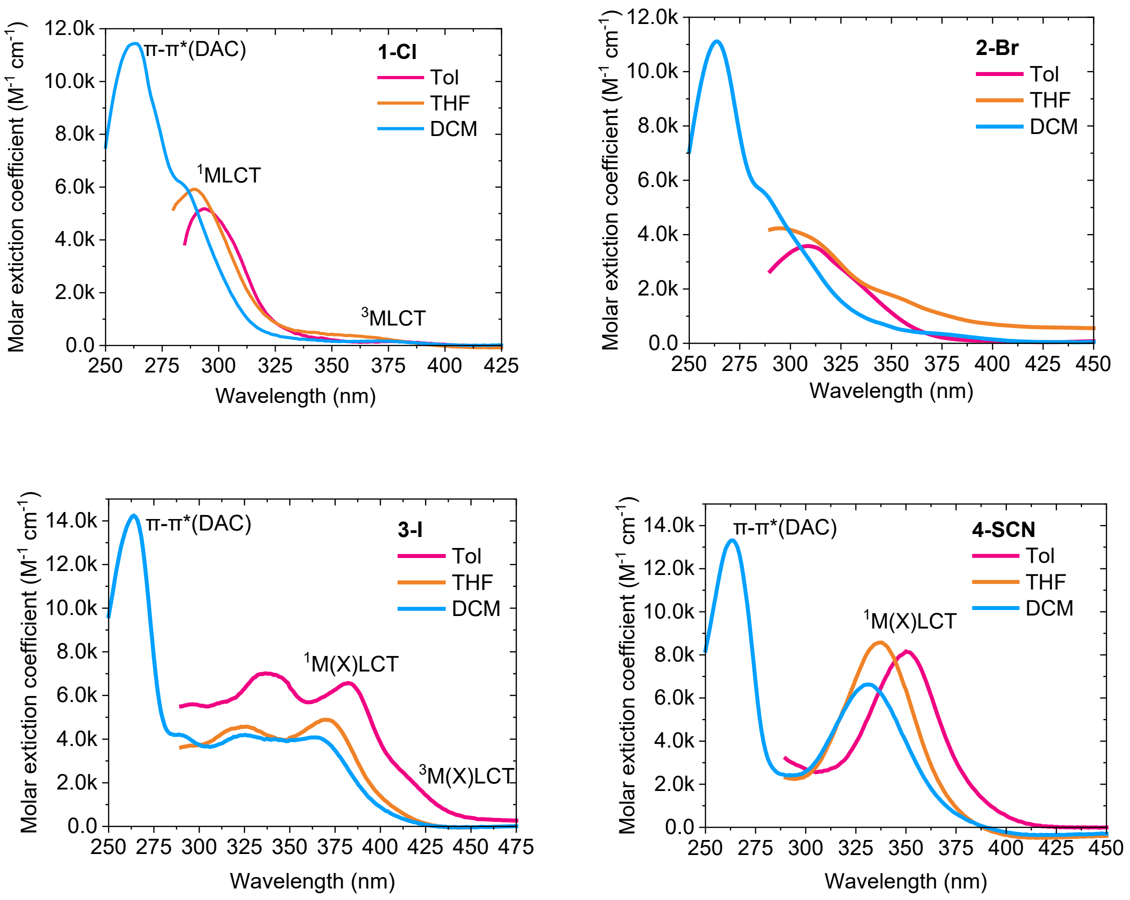

Figure S5. UV-vis spectra for gold complexes **1-Cl** (top left), **2-Br** (top right), **3-I** (bottom left), **4-SCN** (bottom right) in CH_2Cl_2, THF and Toluene solutions at 295 K.

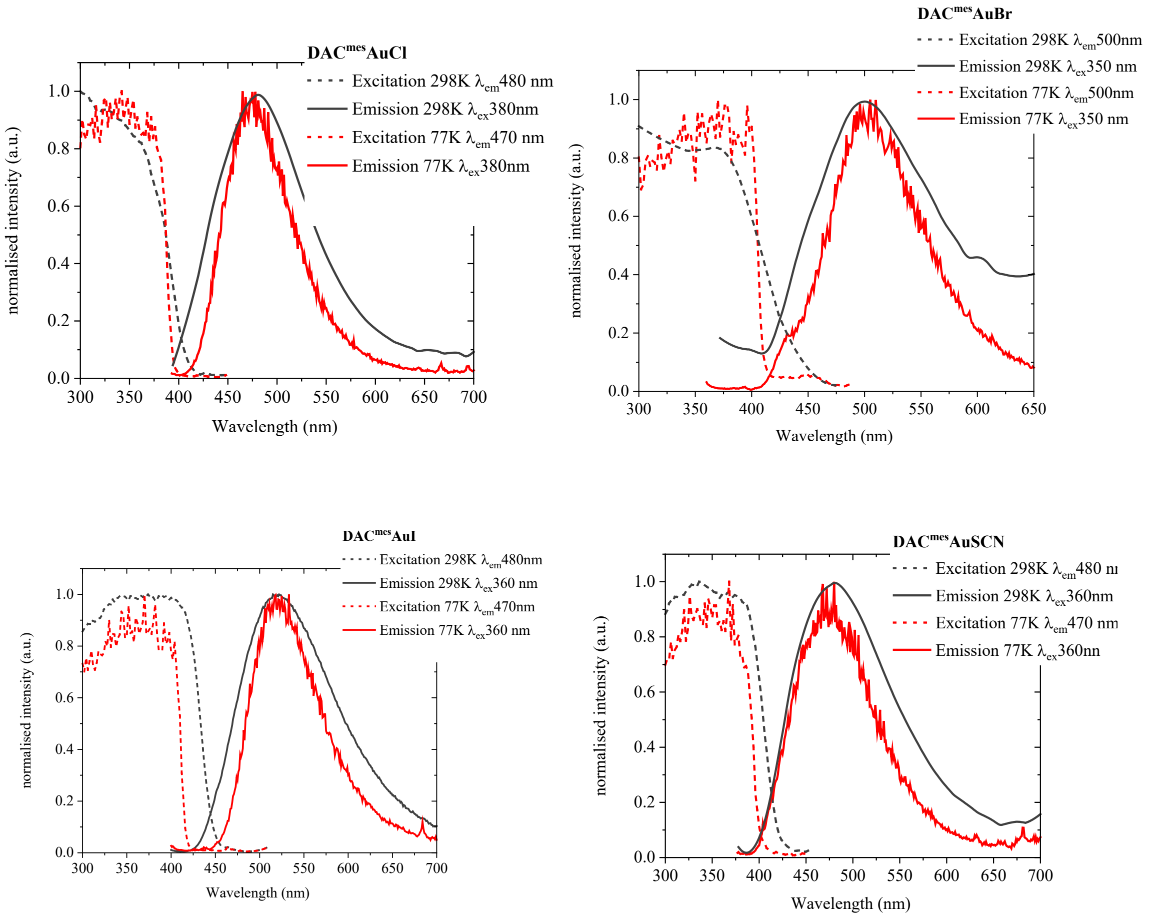

Figure S6. Excitation and emission spectra for gold complexes **1-Cl** (top left), **2-Br** (top right), **3-I** (bottom left), **4-SCN** (bottom right) in the solid state at 298 and 77 K.

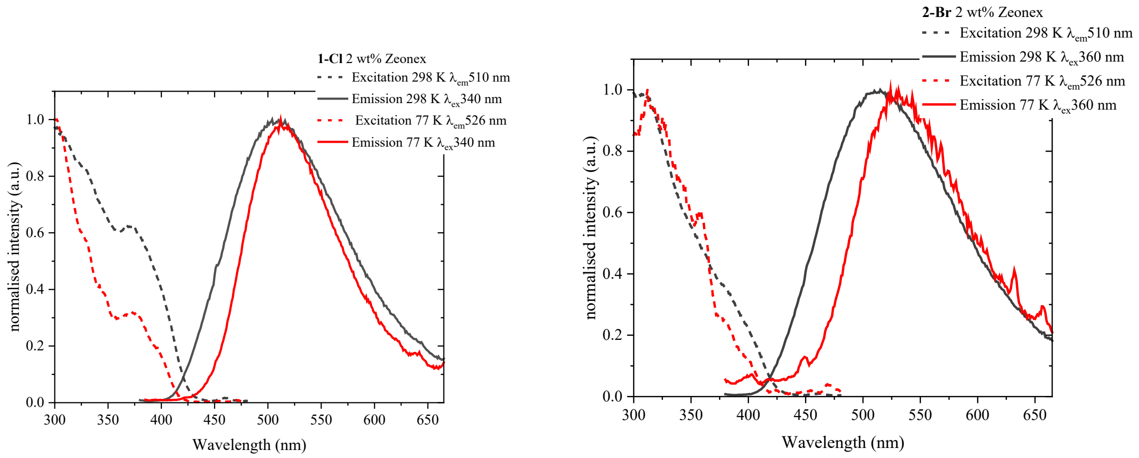

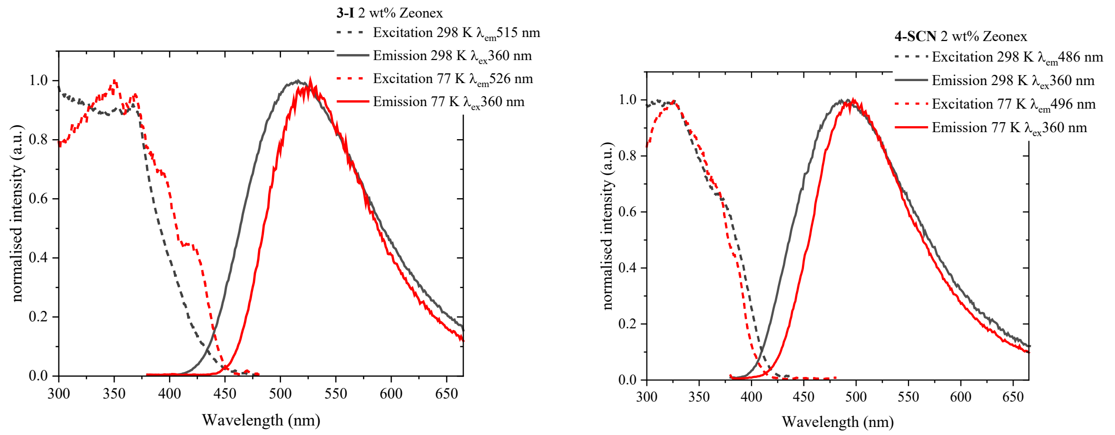

Figure S7. Excitation and emission spectra for gold complexes **1-Cl** (top left), **2-Br** (top right), **3-I** (bottom left), **4-SCN** (bottom right) in 2 wt% Zeonex matrix

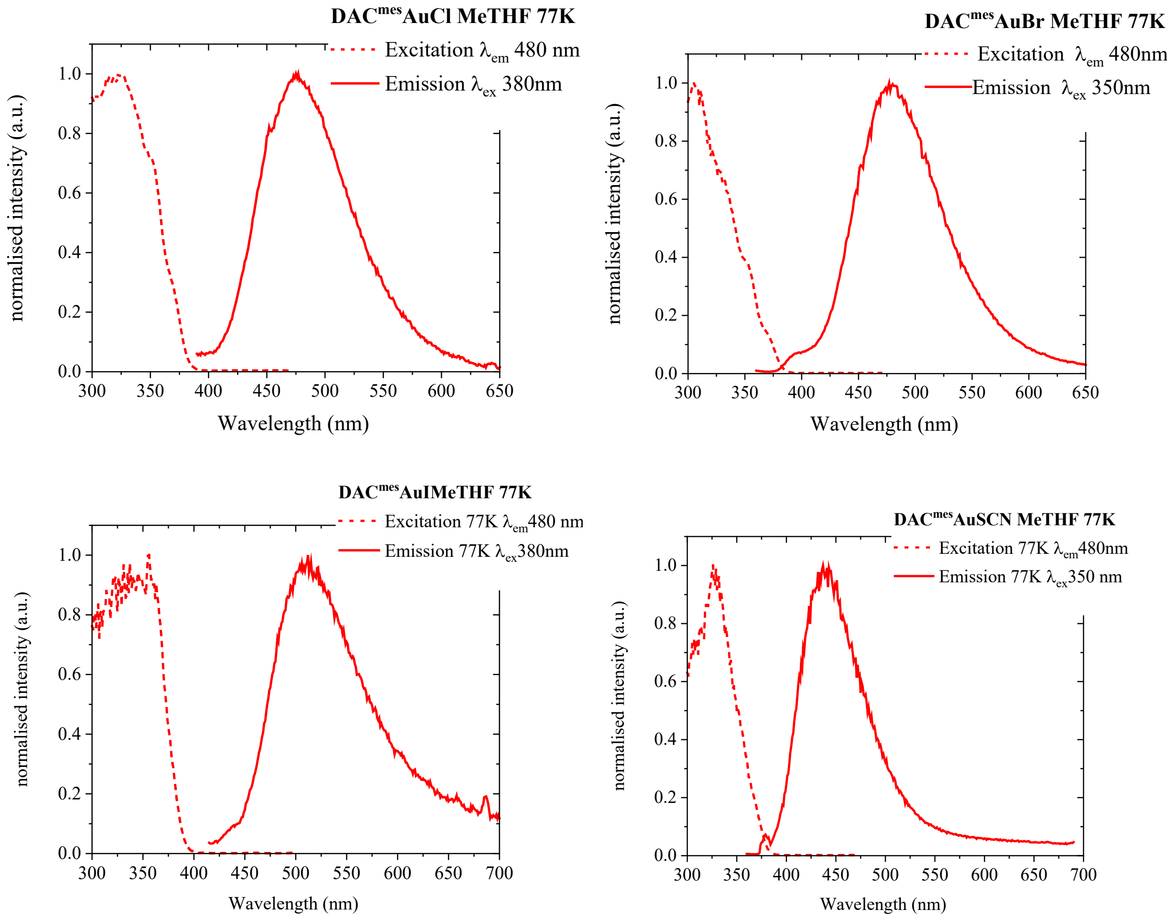

Figure S8. Excitation and emission spectra for gold complexes **1-Cl** (top left), **2-Br** (top right), **3-I** (bottom left), **4-SCN** (bottom right) in frozen MeTHF glass at 77 K.

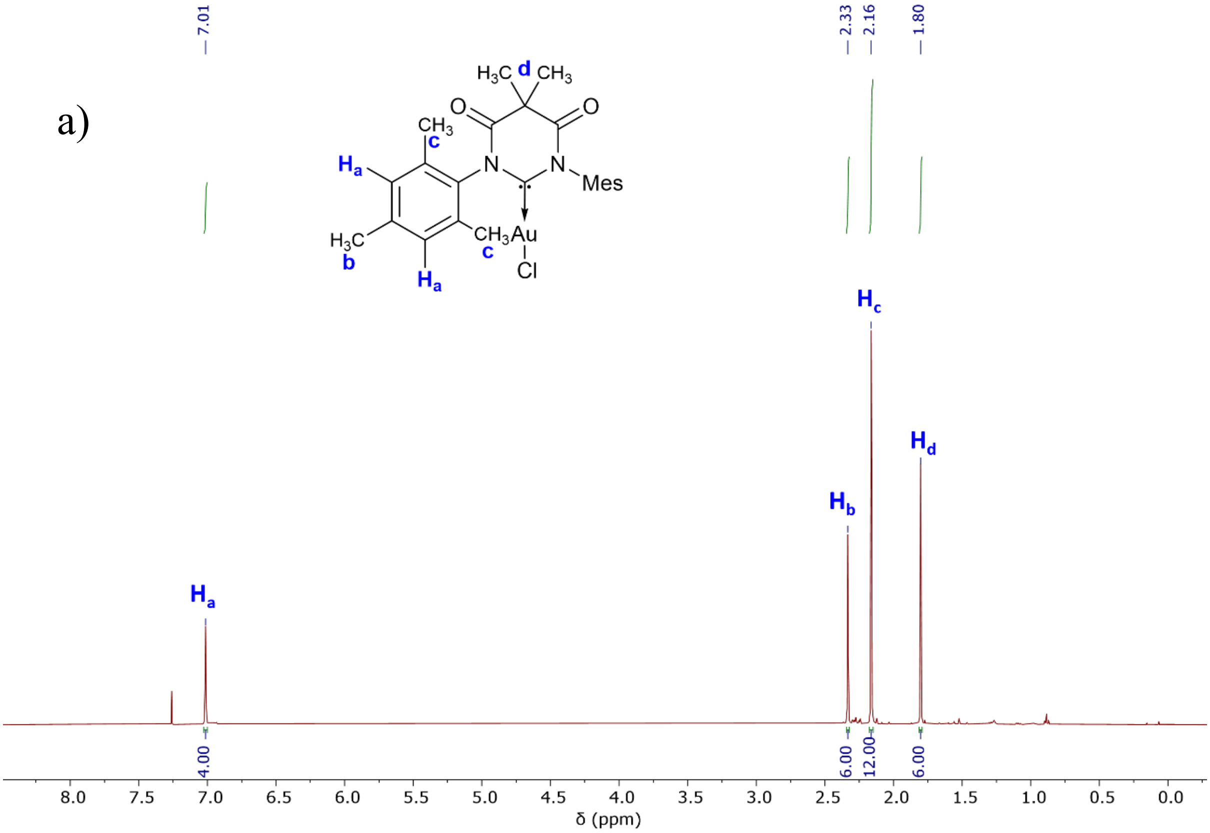

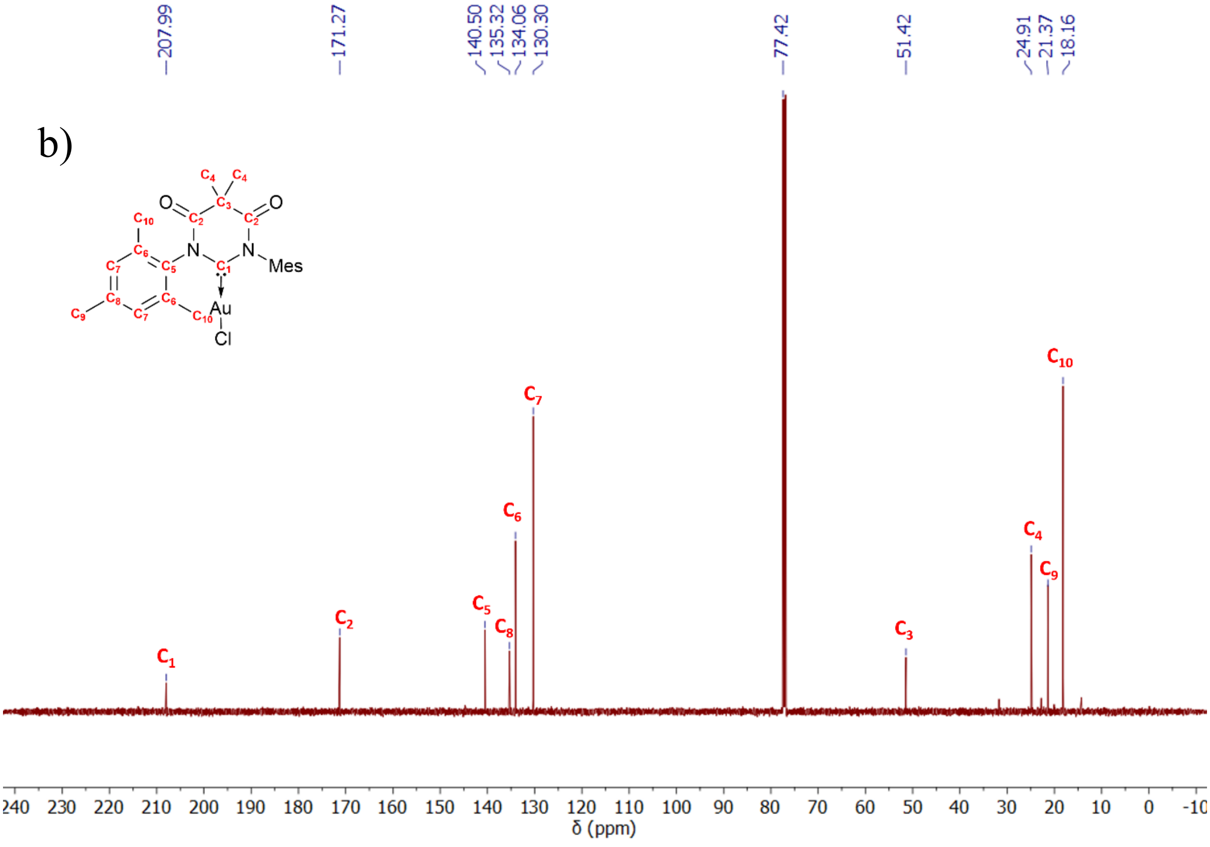

Figure S9. a) ^{1}H NMR spectrum of DAC-Au-Cl (**1-Cl**) (500 MHz, $CDCl_3$); b) ^{13}C NMR spectrum of DAC-Au-Cl (**1-Cl**) (125 MHz, $CDCl_3$).

a)

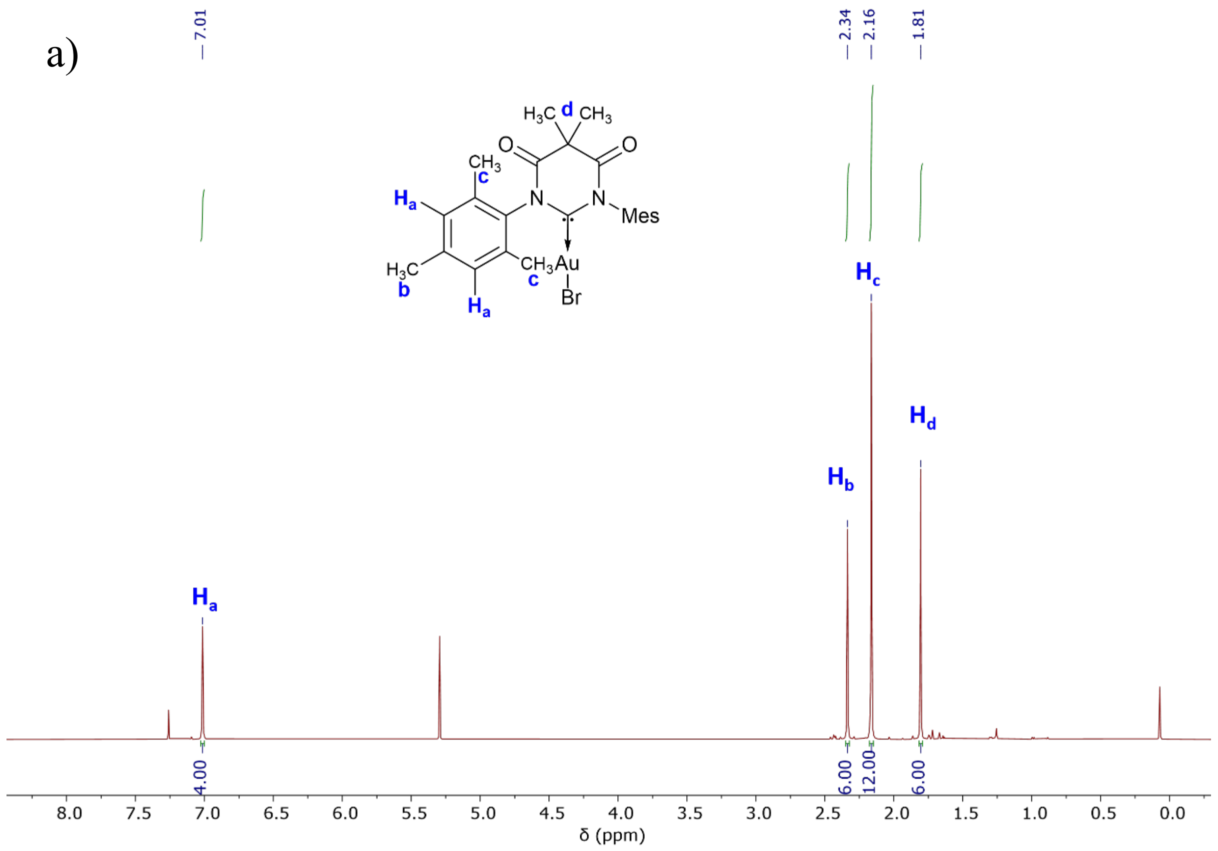

b)

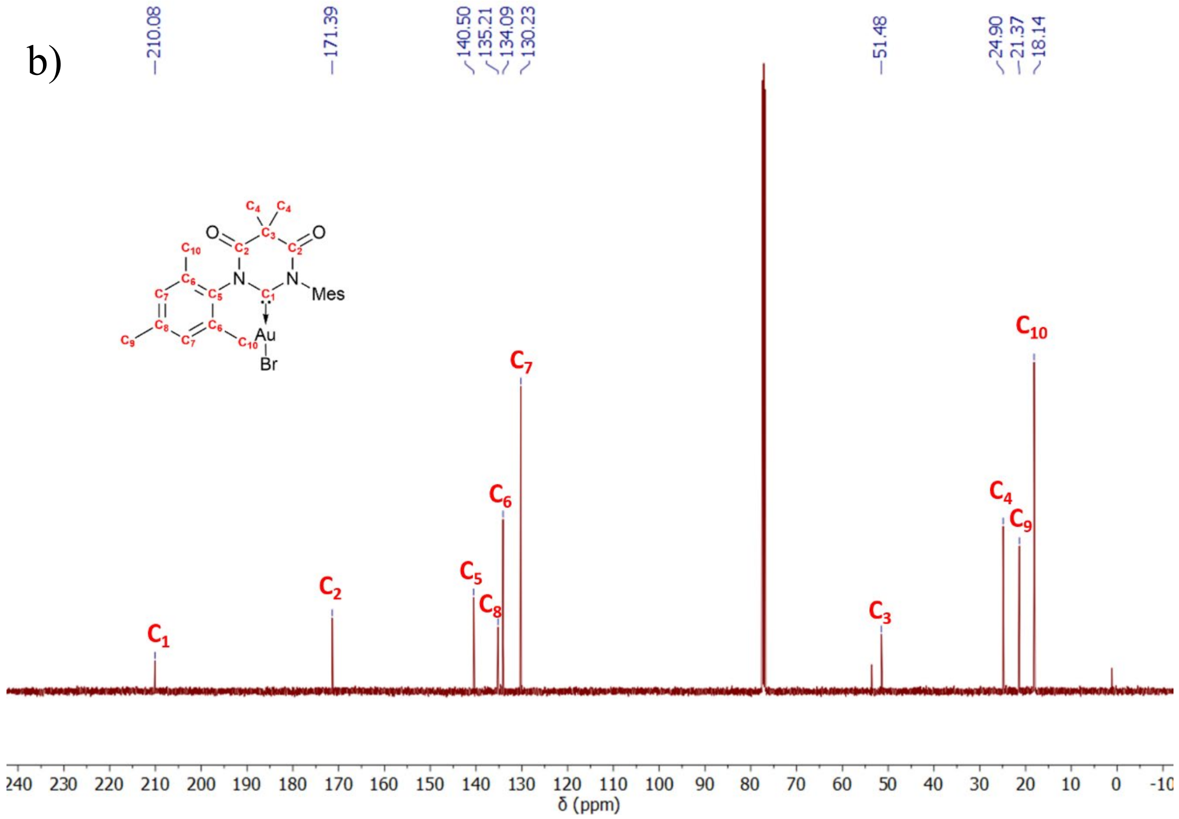

Figure S10. a) ^{1}H NMR spectrum of DAC-Au-Br (**2-Br**) (500 MHz, $CDCl_3$); b) ^{13}C NMR spectrum of DAC-Au-Br (**2-Br**) (125 MHz, $CDCl_3$).

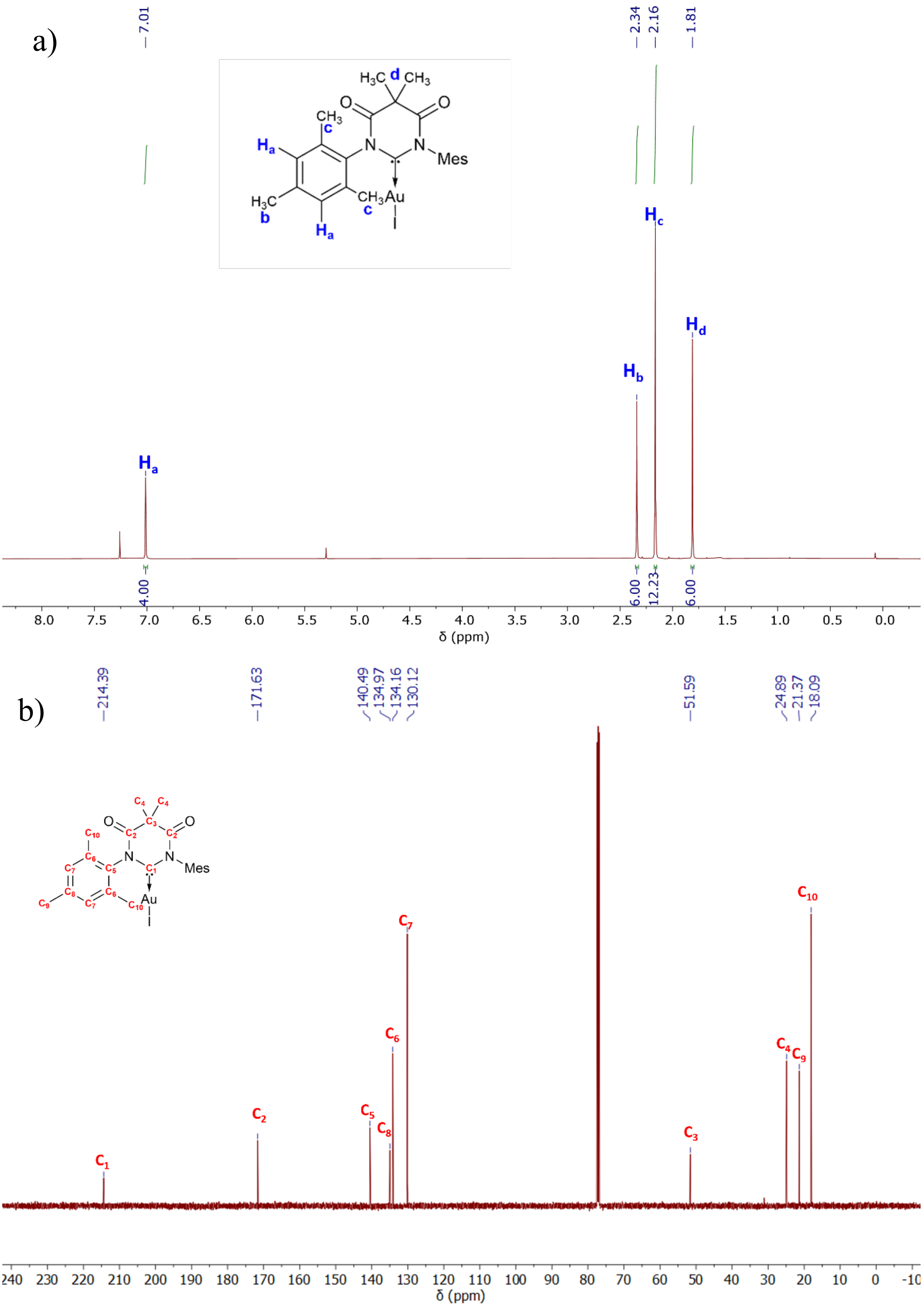

Figure S11. a) ^{1}H NMR spectrum of DAC-Au-I (**3-I**) (500 MHz, $CDCl_3$); b) ^{13}C NMR spectrum of DAC-Au-I (**3-I**) (125 MHz, $CDCl_3$).

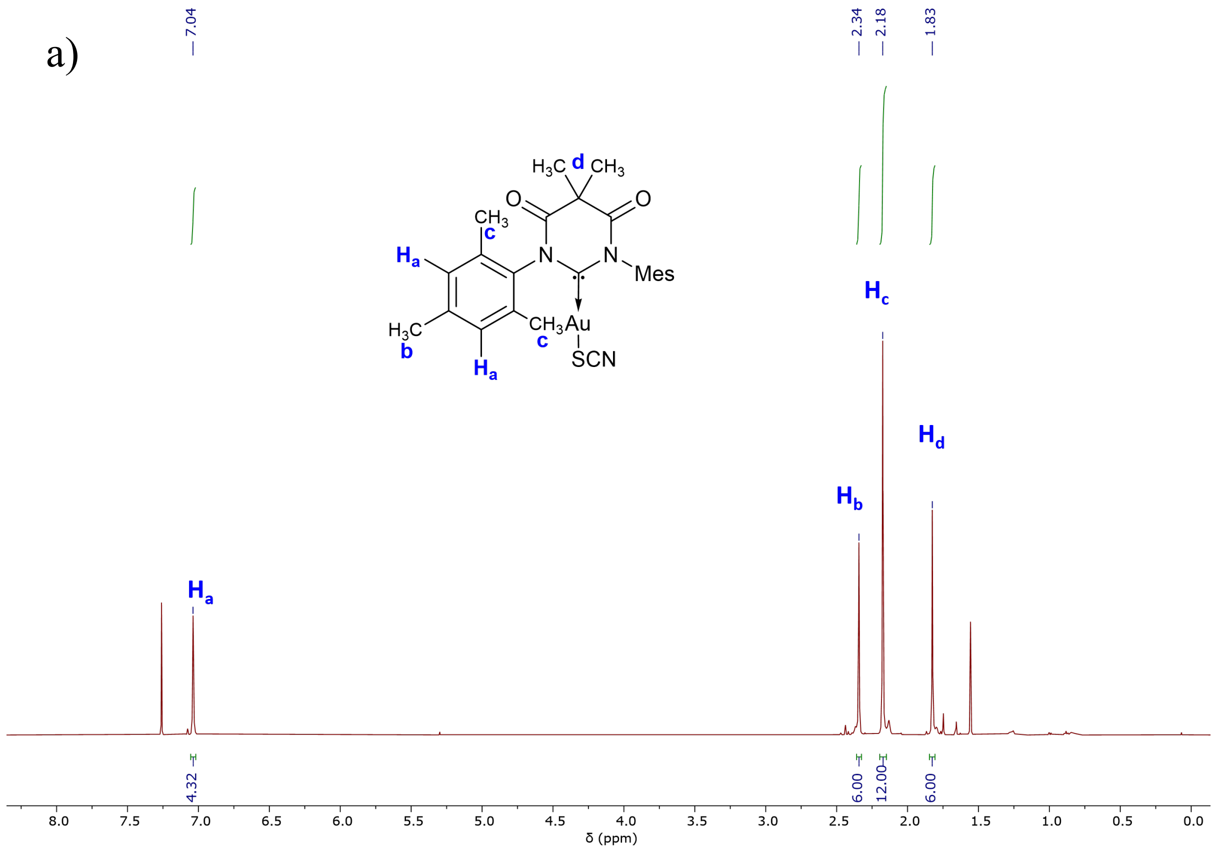

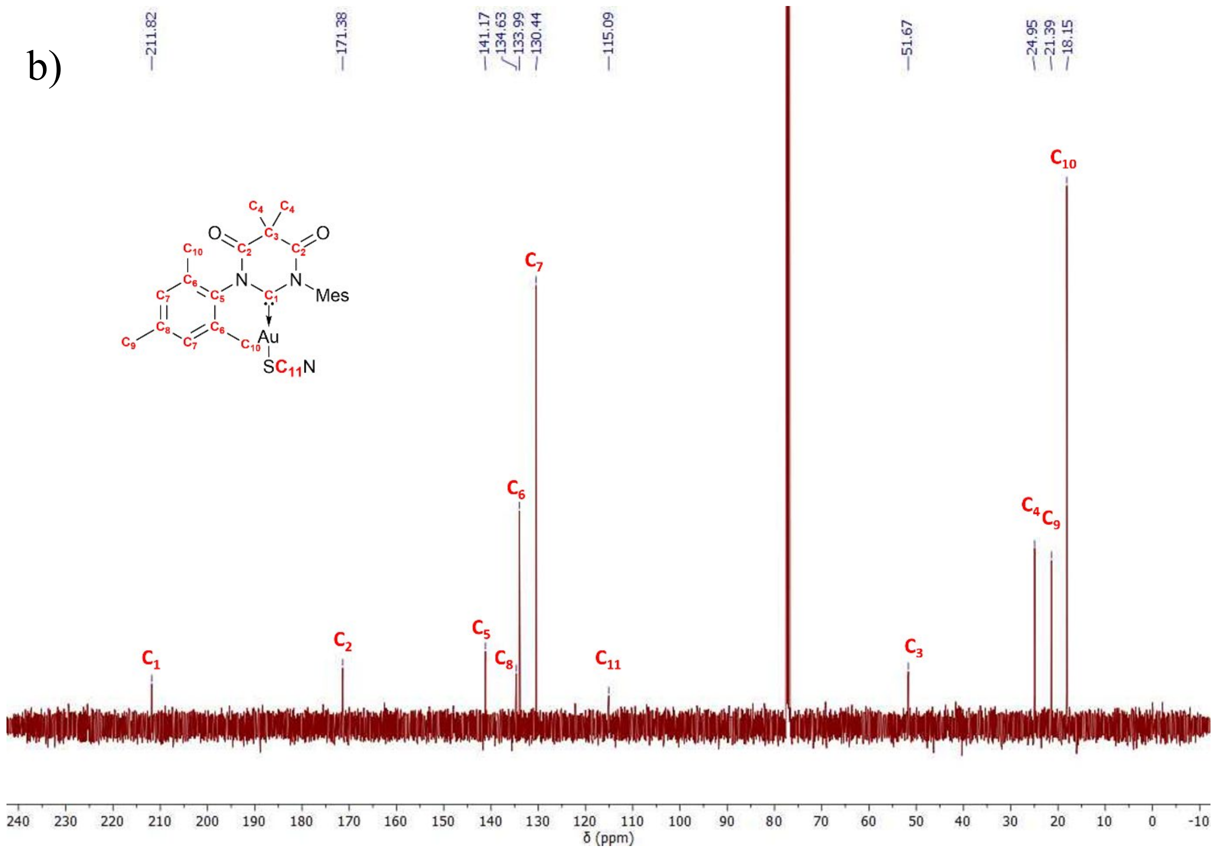

Figure S12. a) ^{1}H NMR spectrum of DAC-Au-SCN (**4-SCN**) (500 MHz, $CDCl_3$); b) ^{13}C NMR spectrum of DAC-Au-SCN (**4-SCN**) (125 MHz, $CDCl_3$).

a)

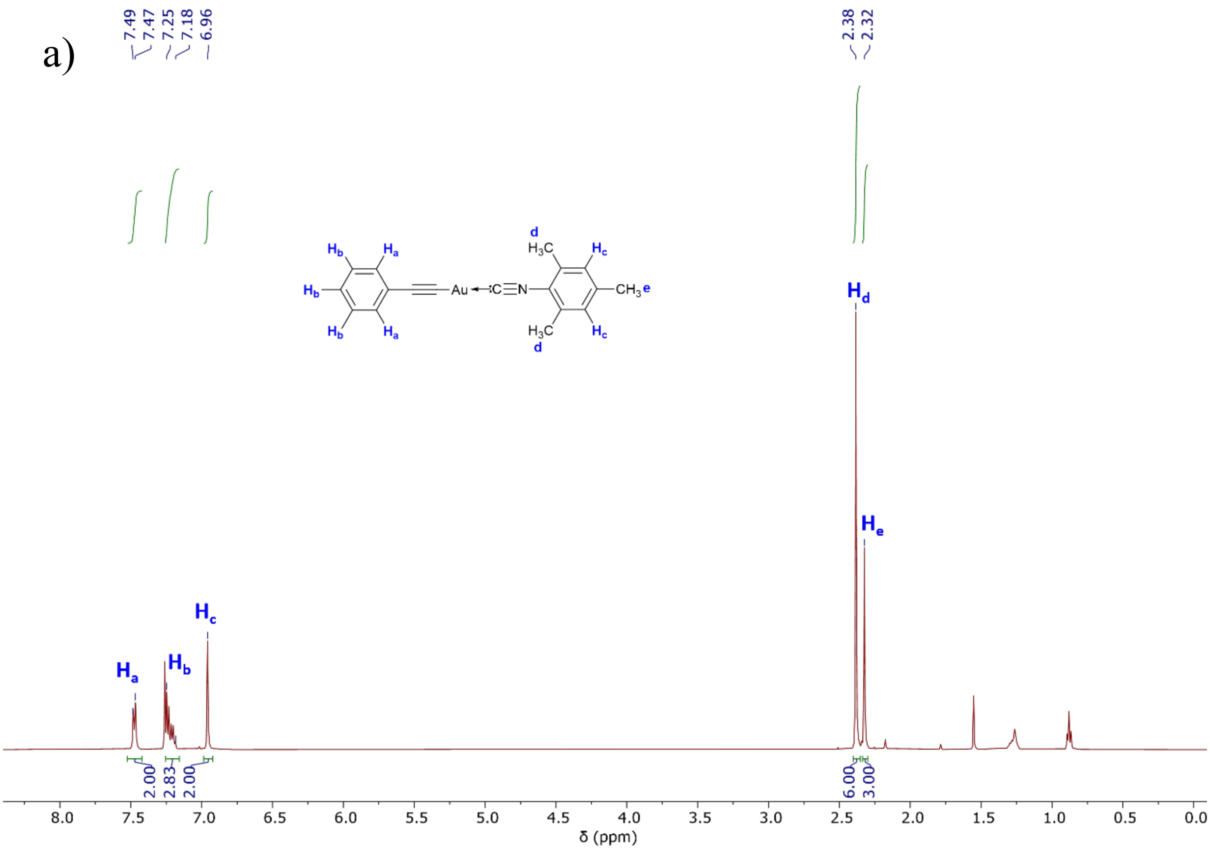

b)

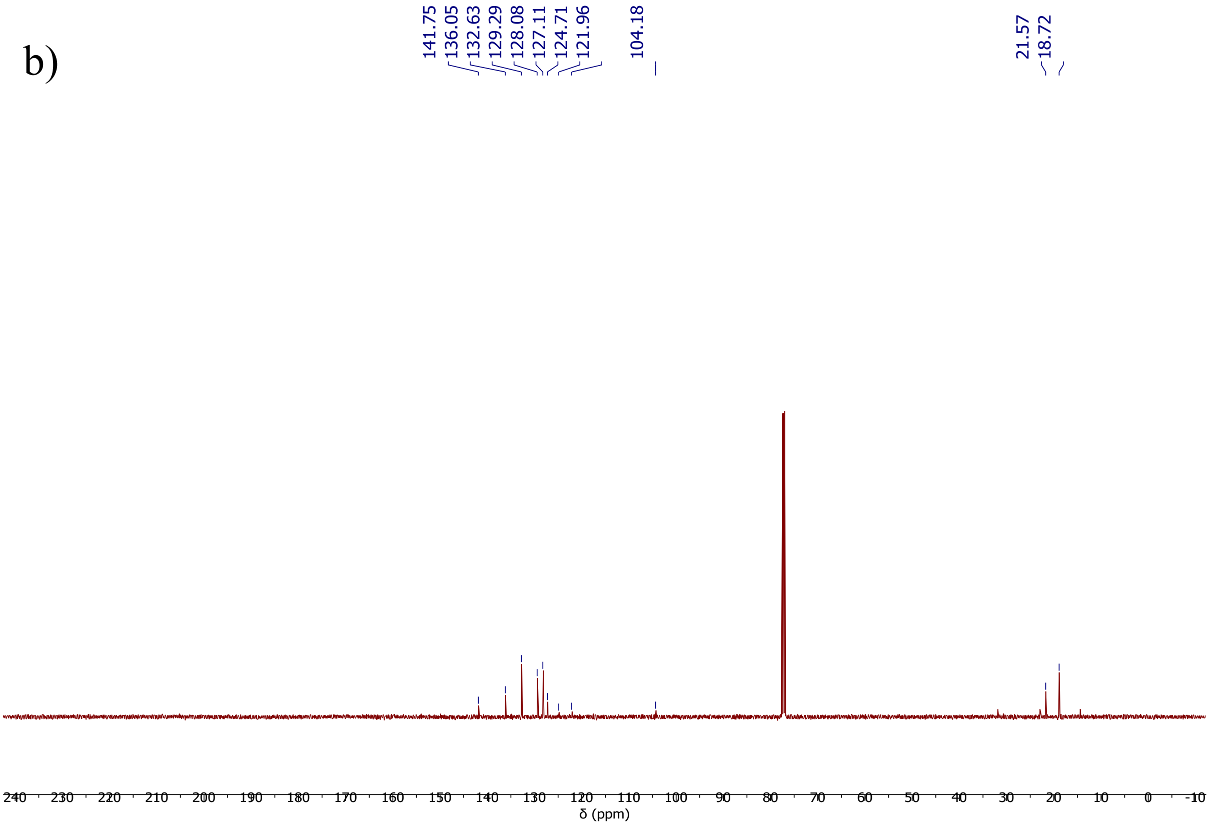

Figure S13. a) ^{1}H NMR spectrum of MesC≡NAuC≡CPh (**5**) (500 MHz, $CDCl_3$); b) ^{13}C NMR spectrum of MesC≡NAuC≡CPh (**5**) (125 MHz, $CDCl_3$).

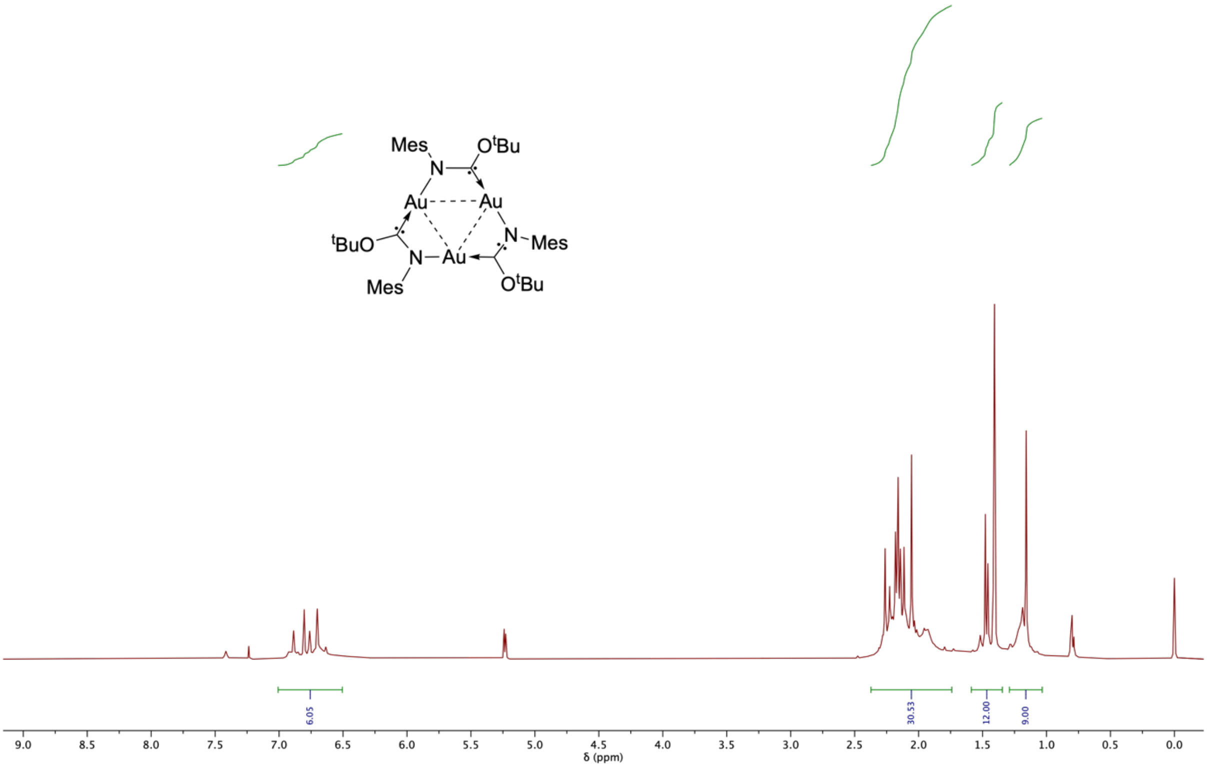

Figure S14. ^{1}H NMR spectrum of [Au(N(Mes)C=OC[t]Bu)3] (**6**) (500 MHz, CD_2Cl_2)

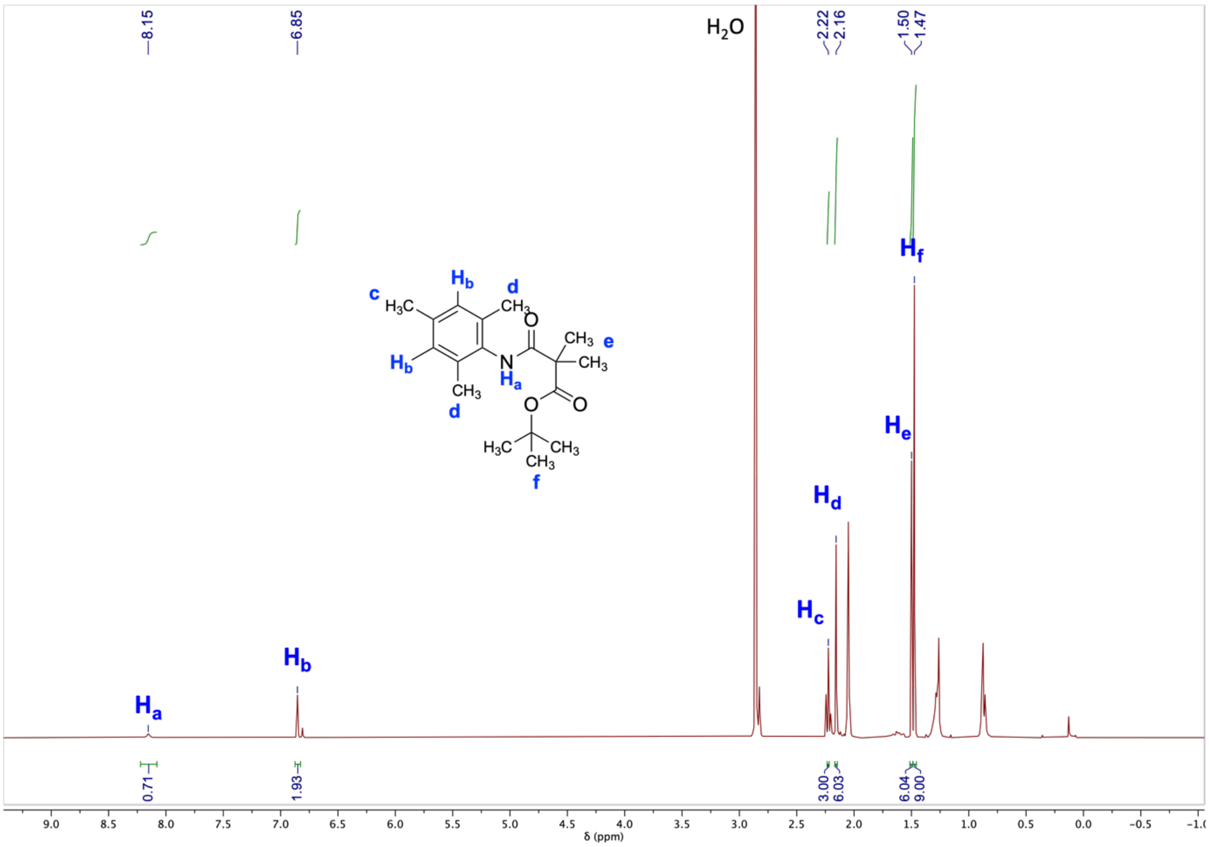

Figure S15. ^{1}H NMR spectrum of DAC backbone fragment (**7**) in $(CD_3)_2CO$

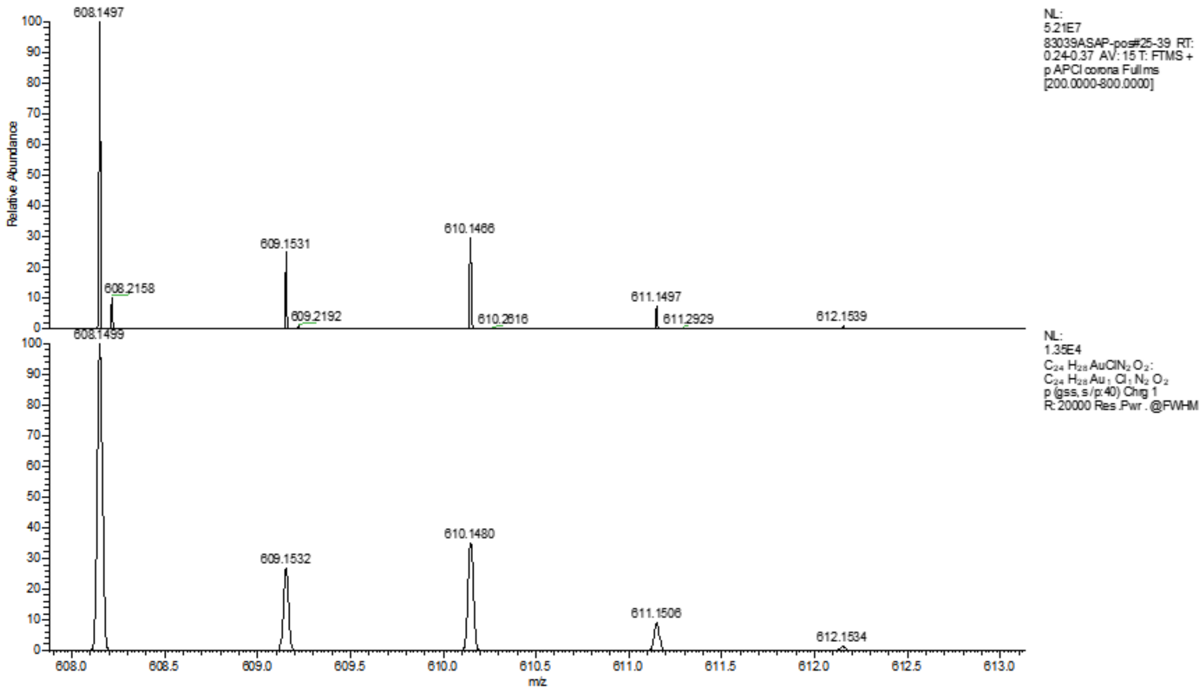

Figure S16. APCI spectrum of **1-Cl**

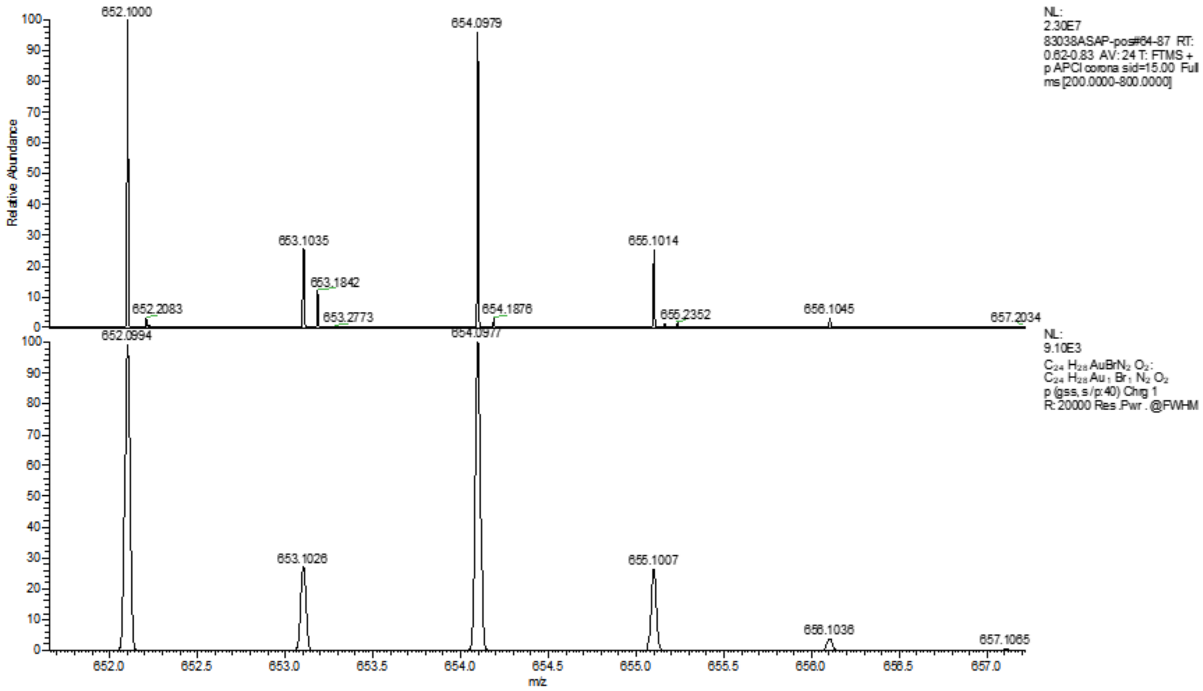

Figure S17. APCI spectrum **2-Br**

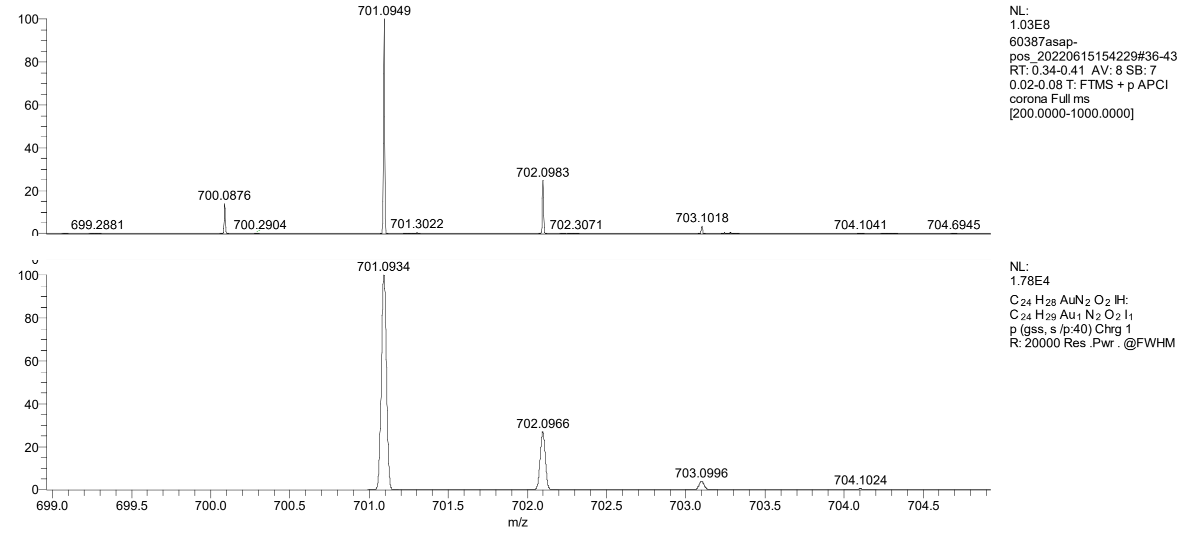

Figure S18. APCI spectrum **3-I**

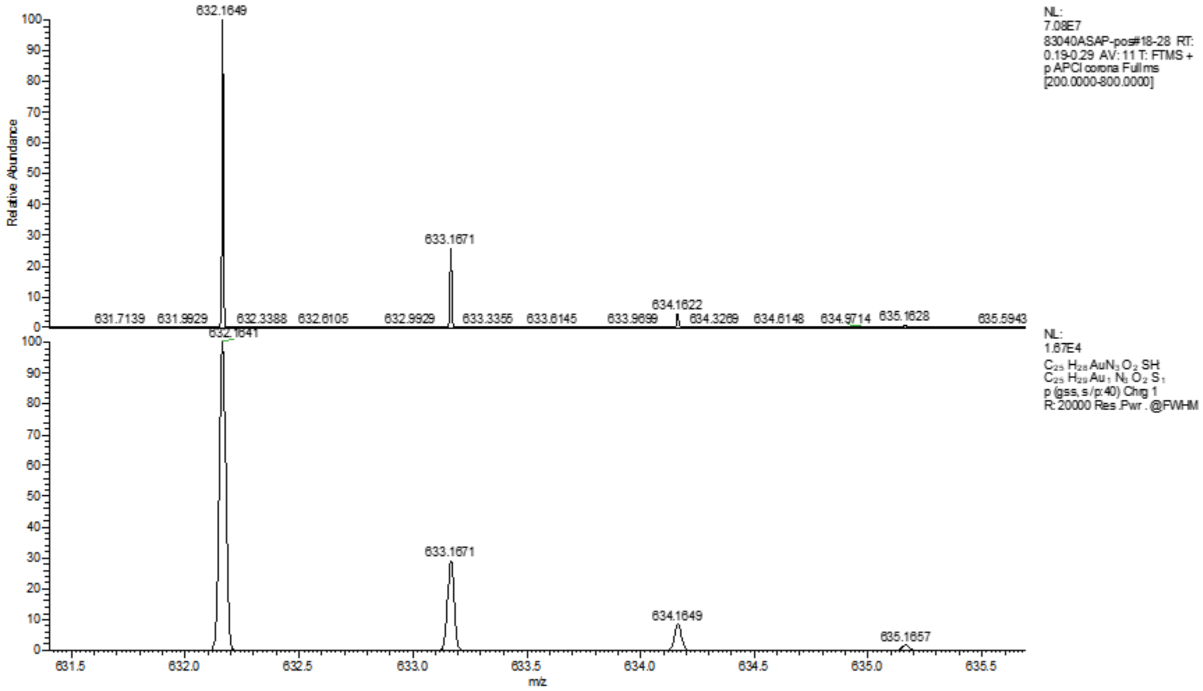

Figure S19. APCI spectrum **4-SCN**

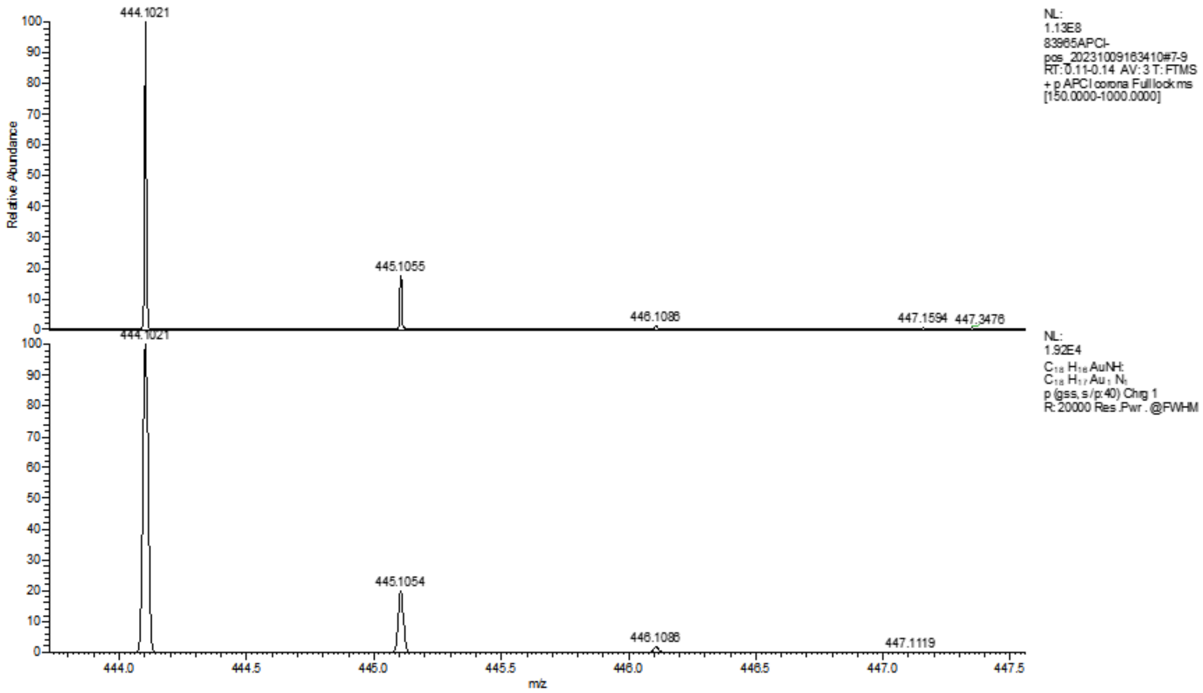

Figure S20. APCI spectrum (MesC≡N)Au(C≡CPh) (**5**)

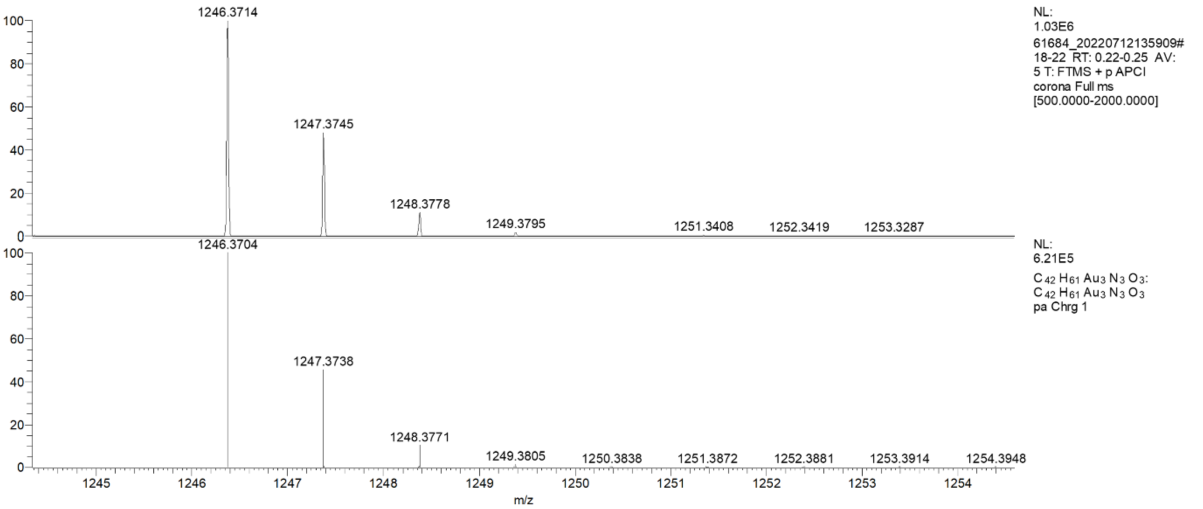

Figure S21. APCI spectrum [Au(N(Mes)C=OC^{t}Bu)]$_3$ (**6**)

Table S3. HOMO and LUMO isosurface plots, HOMO-LUMO overlap integrals, and metal atom contributions to the orbitals in the optimized S_0 geometry.

	HOMO	LUMO
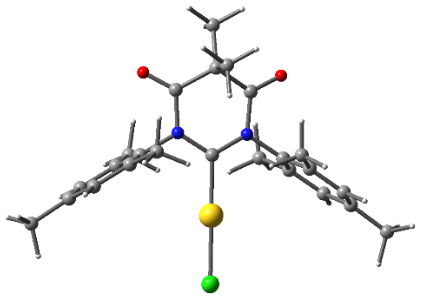<br>**1-Cl**<br>Overlap integral: 0.23	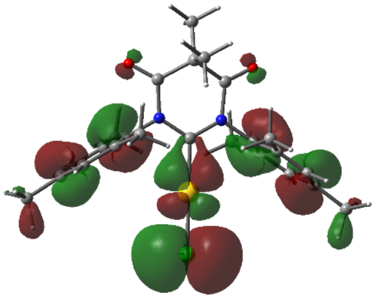<br>16.7%Au	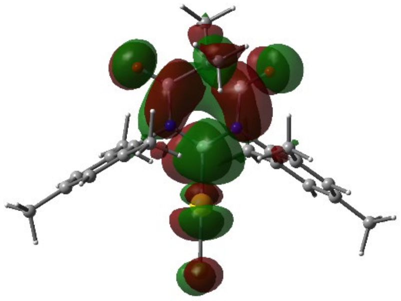<br>5.7%Au
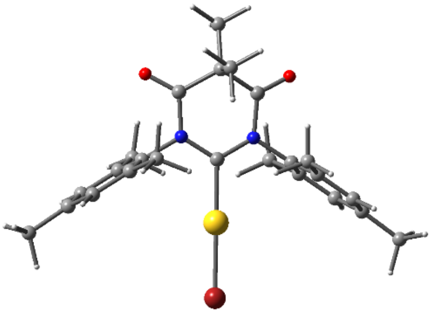<br>**2-B**rOverlap integral: 0.20	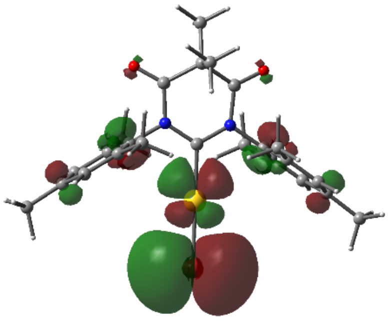<br>13.6%Au	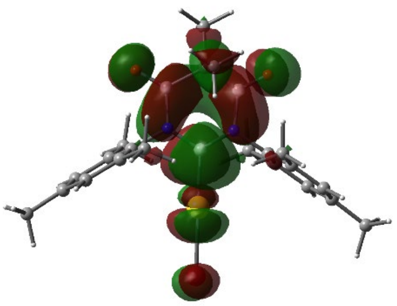<br>5.8%Au
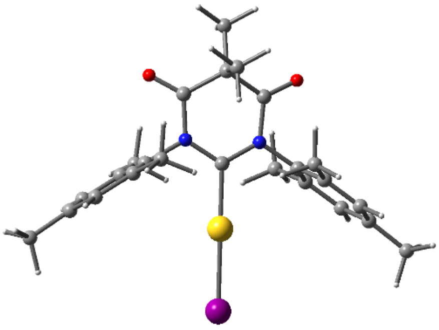<br>**3-I**Overlap integral: 0.18	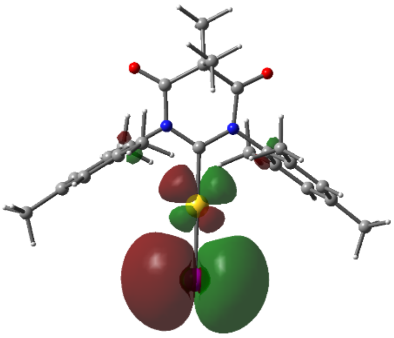<br>8.6%Au	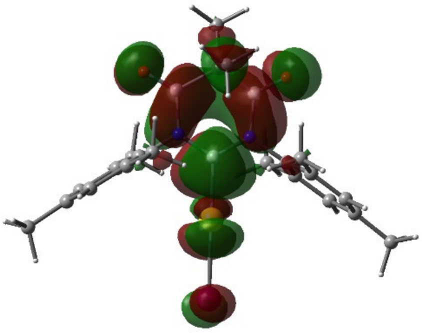<br>5.9%Au
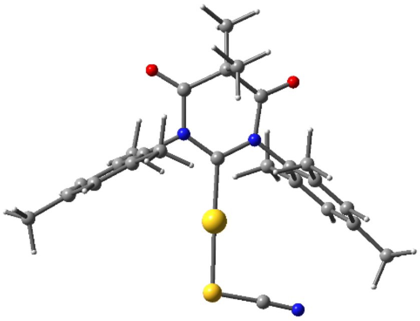<br>**4-SCN**<br>Overlap integral: 0.35	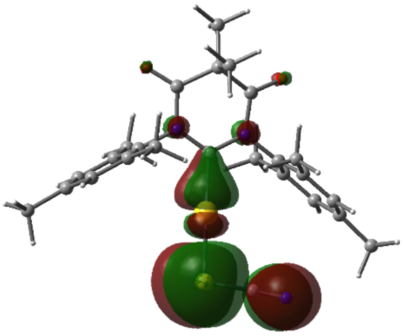<br>8.5%Au	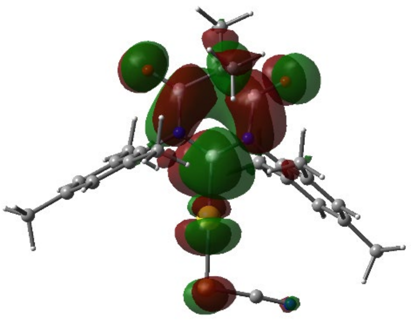<br>5.5%Au

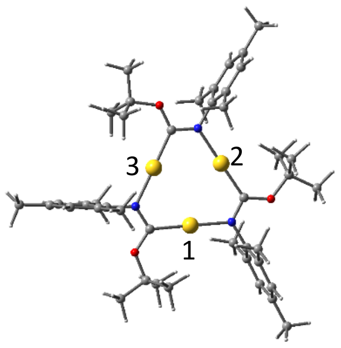<br>**6**<br>Overlap integral: 0.65	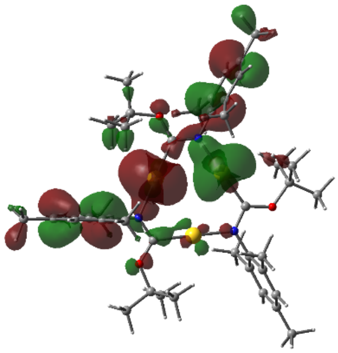<br>0.9%Au1<br>15.0%Au2<br>22.6%Au3	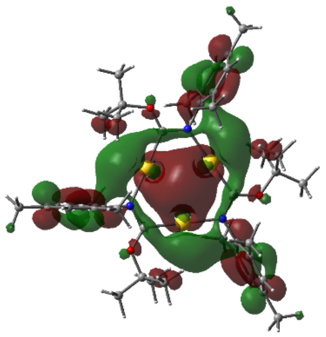<br>16.9%Au1<br>16.9%Au2<br>16.9%Au3

Table S4. Dipole moments for S_0 and lowest singlet vertical excitations in the optimized S_0 geometry.

	S_0	$S_1@S_0$
**1-Cl**	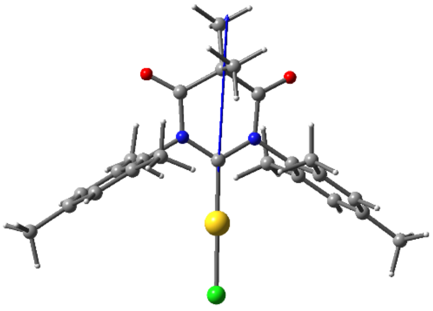<br>5.2D	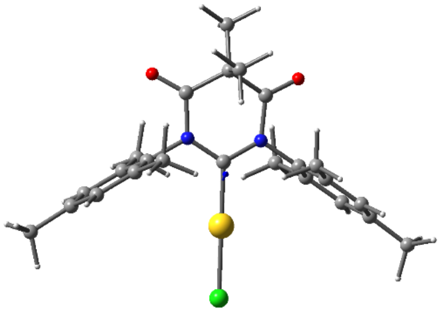<br>0.6D
**2-B**r	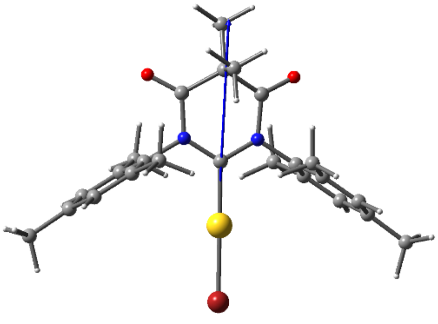<br>5.3D	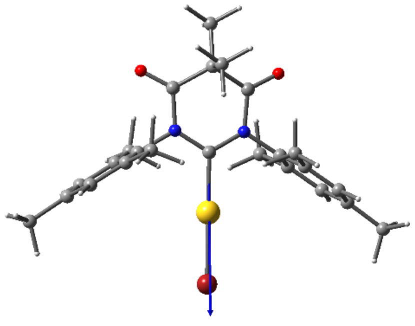<br>(-)4.7D

**3-I**	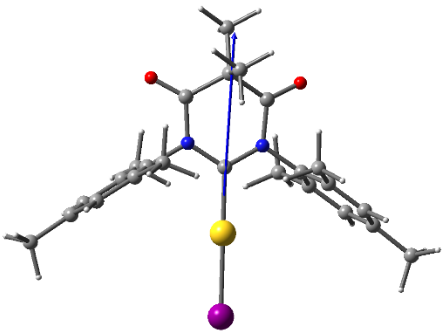 5.2D	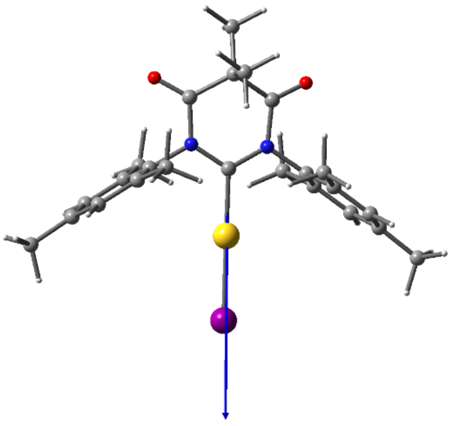 (-)6.5D
**4-SCN**	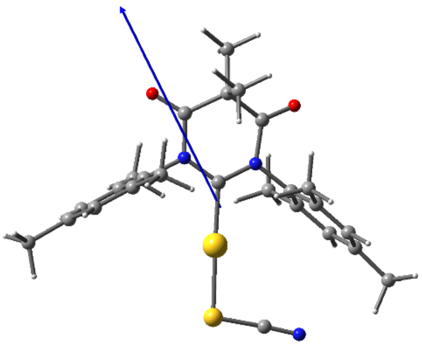 7.3D	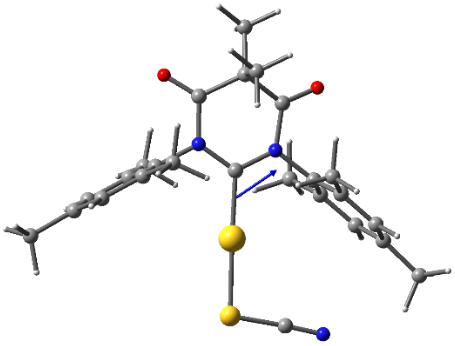 1.4D

Table S5. Vertical excitations, their character, and S_0-S_1 oscillator strength coefficients.

	Excitation energy	Character	Oscillator strength
**1-Cl**	S_1: 3.40eV = 364nm	HOMO-1 – LUMO (86%)	0.0030
	S_2: 3.56eV = 348nm	HOMO – LUMO (92%)	0.0000
	S_6: 4.23eV = 293nm	HOMO-2 – LUMO (70%)<br>HOMO-4 – LUMO (20%)	0.1531
	T_1: 3.18eV = 390nm	HOMO-1 – LUMO (76%)	
	T_2: 3.51eV = 353nm	HOMO – LUMO (90%)	
**2-Br**	S_1: 3.37eV = 368nm	HOMO – LUMO (94%)	0.0000
	S_3: 3.88eV = 320nm	HOMO-1 – LUMO (95%)	0.1735
	T_1: 3.17eV = 391nm	HOMO-2 – LUMO (73%)	
	T_2: 3.33eV = 373nm	HOMO – LUMO (92%)	
**3-I**	S_1: 3.00eV = 413nm	HOMO – LUMO (96%)	0.0000
	S_2: 3.38eV = 366nm	HOMO-1 – LUMO (92%)	0.1514
	T_1: 2.96eV = 418nm	HOMO – LUMO (95%)	
	T_2: 2.97eV = 417nm	HOMO-1 – LUMO (95%)	
**4-SCN**	S_1: 3.38eV = 367nm	HOMO-1 – LUMO (88%)	0.0022
	S_2: 3.57eV = 348nm	HOMO – LUMO (96%)	0.1622
	T_1: 3.17eV = 392nm	HOMO – LUMO (92%)	
	T_2: 3.21eV = 386nm	HOMO-1 – LUMO (67%)	
**6**	S_1: 4.75eV = 261nm	HOMO-1 – LUMO+2 (38%)<br>HOMO – LUMO+1 (38%)	0.0294
	S_2=S_3: 4.81eV = 258nm		0.0000
	S_4=S_5: 5.08eV = 244nm	S_4: HOMO-3 – LUMO (8%)<br>HOMO-1 – LUMO (75%)	0.2508

		HOMO – LUMO (4%)<br>S_5: HOMO-4 – LUMO (8%)<br>HOMO-1 – LUMO (4%)<br>HOMO – LUMO (75%)	0.2507
	T_1=T_2: 3.99eV = 311nm		
	T_3: 4.00eV = 310nm		

Table S6. Natural transition orbitals (NTOs) for vertical excited singlet and triplet states.

		HONTO	LUNTO
**1-Cl**	S_1	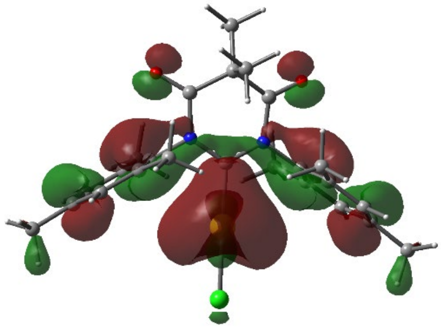	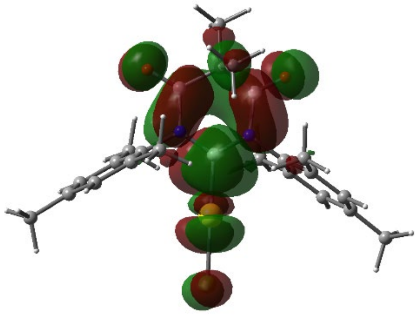
	S_2	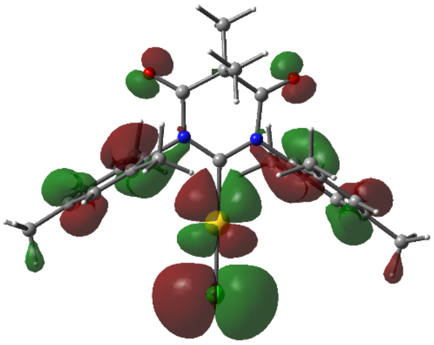	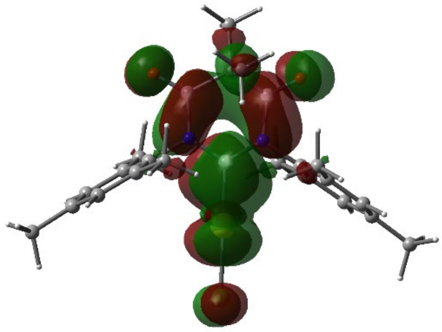
	S_6	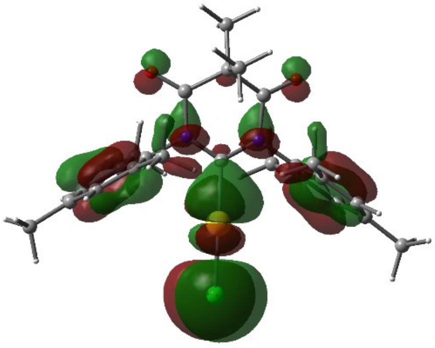	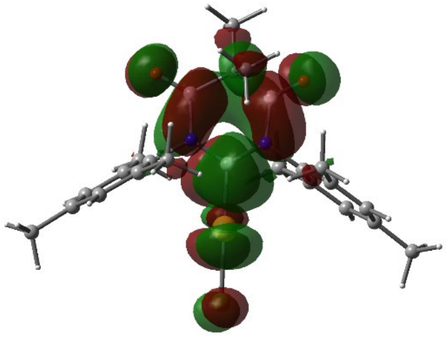
	T_1	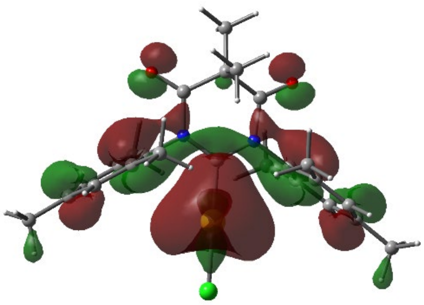	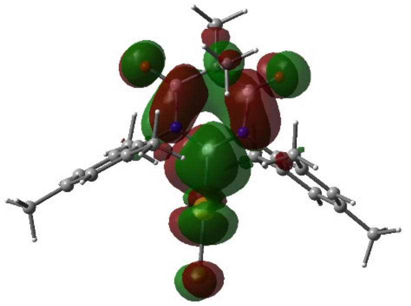

	T_2	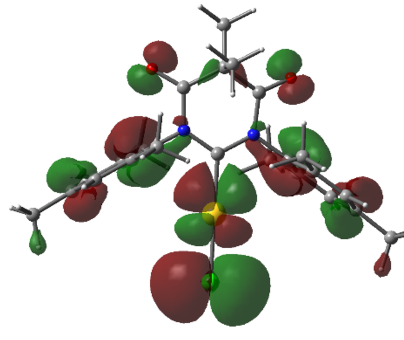	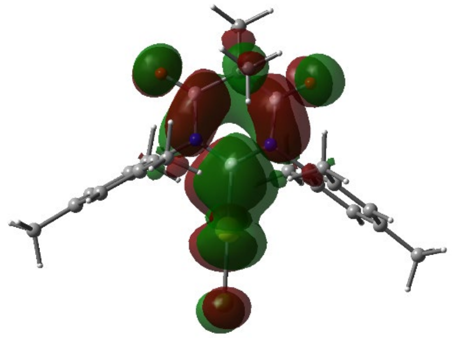
**2-B**r	S_1	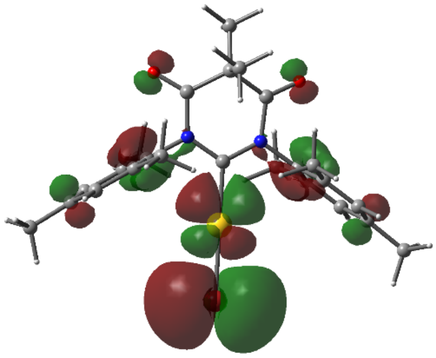	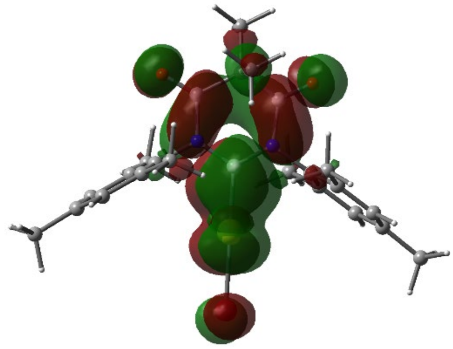
	S_3	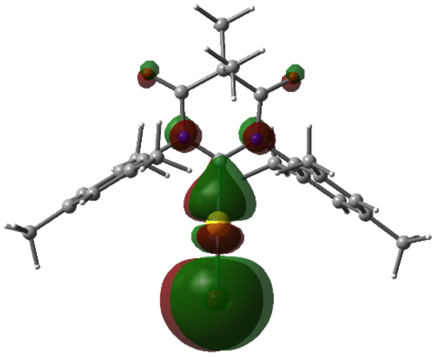	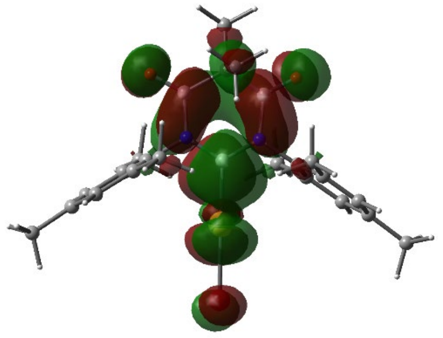
	T_1	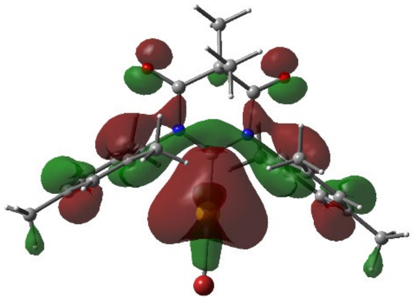	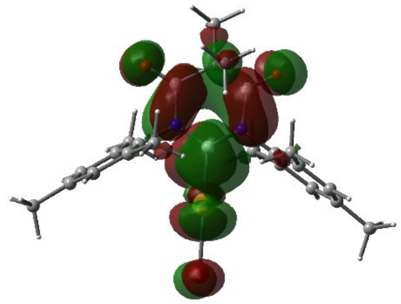
	T_2	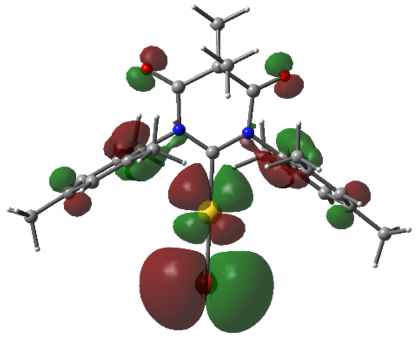	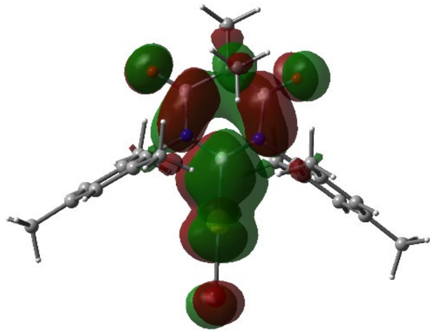

**3-I**	S_1	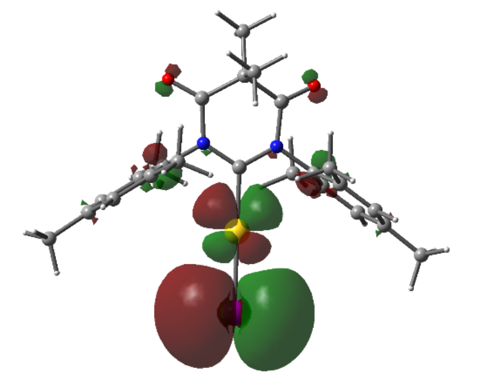	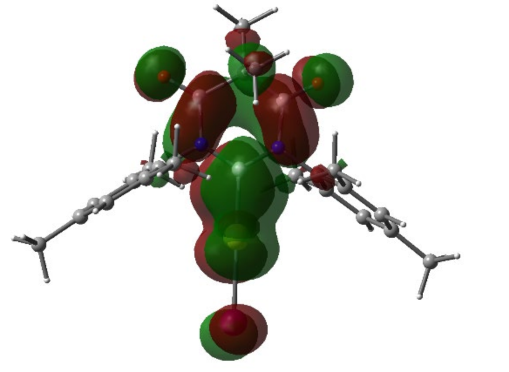
	S_2	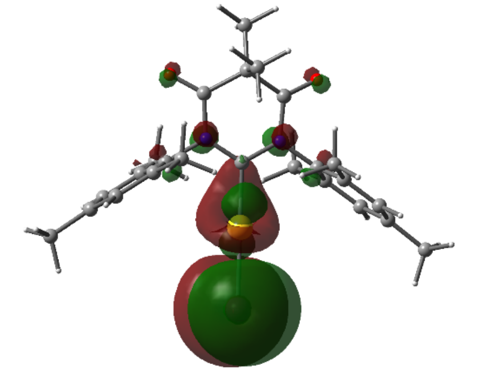	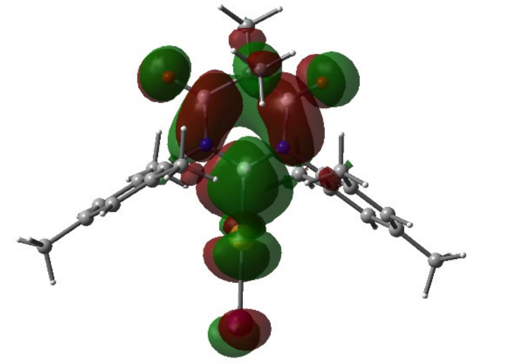
	T_1	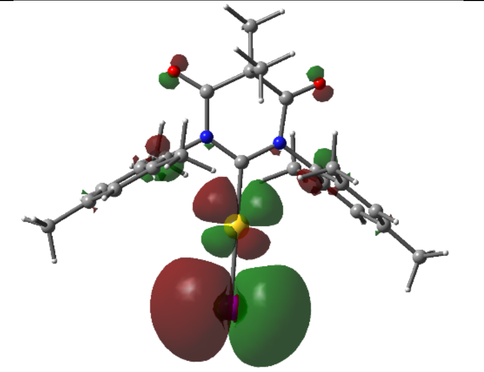	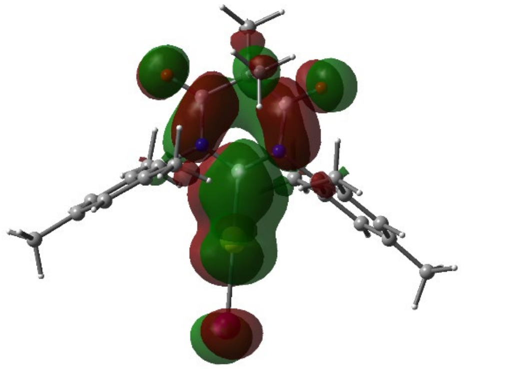
	T_2	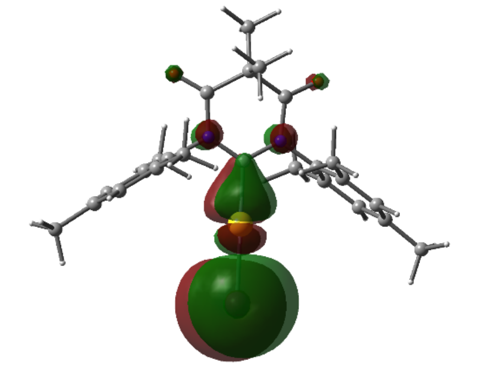	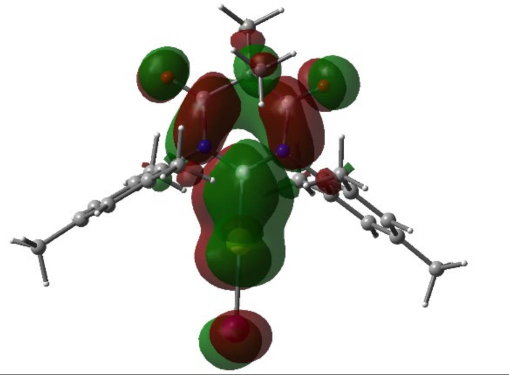

**4-SCN**	S_1	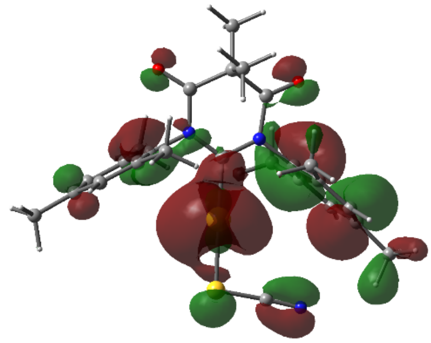	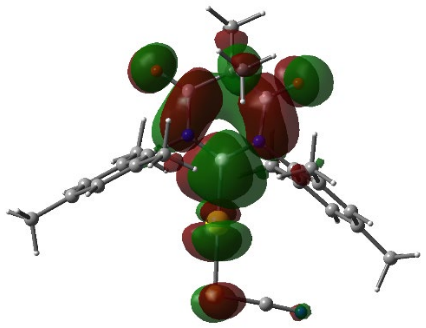
	S_2	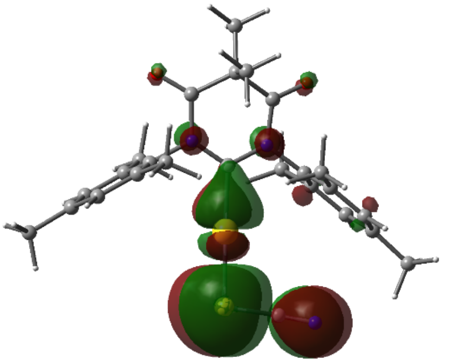	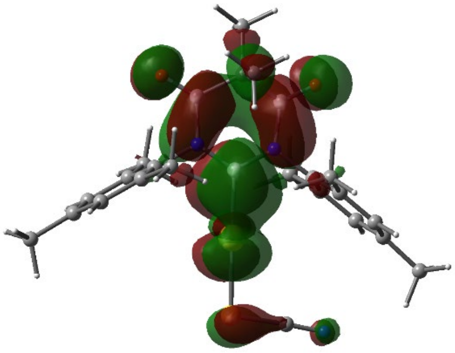
	T_1	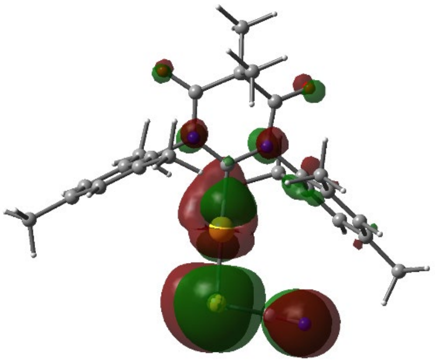	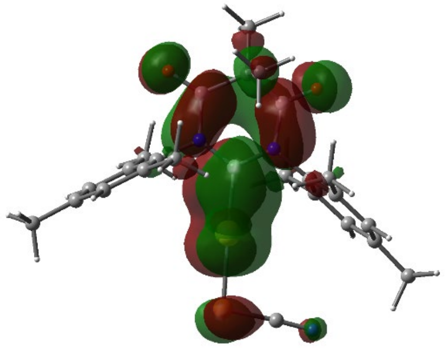
	T_2	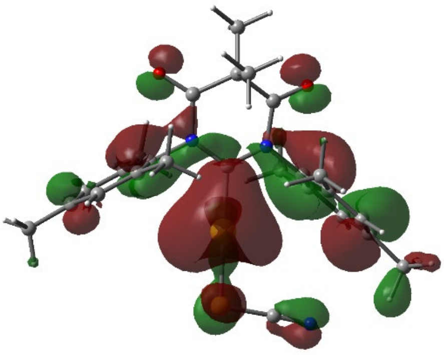	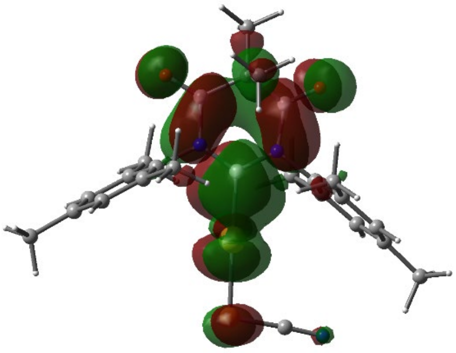
**6**	S_1	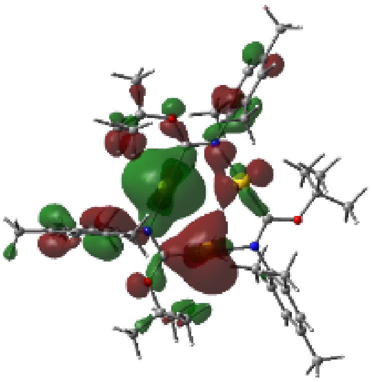	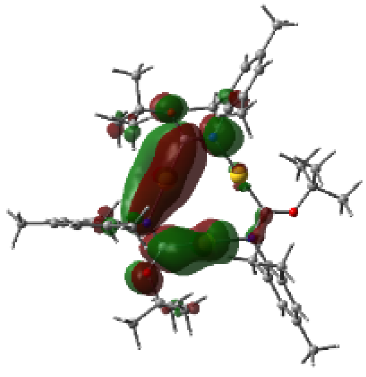

	S_2	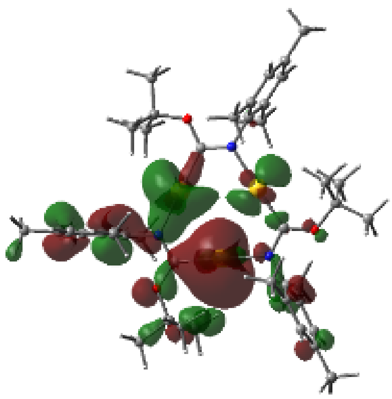	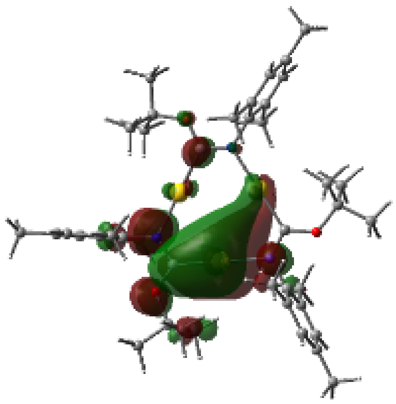
	S_3	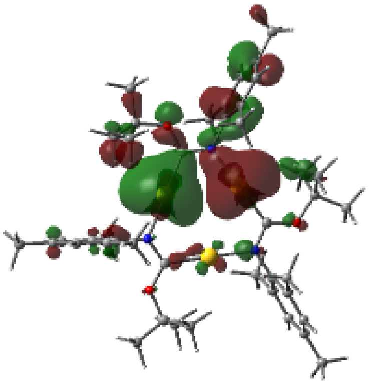	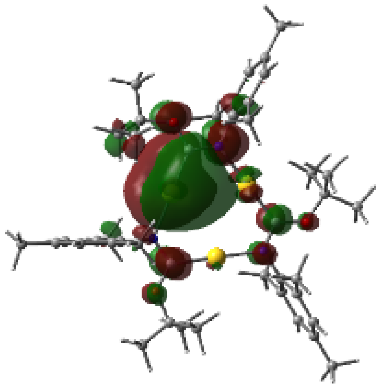
	S_4	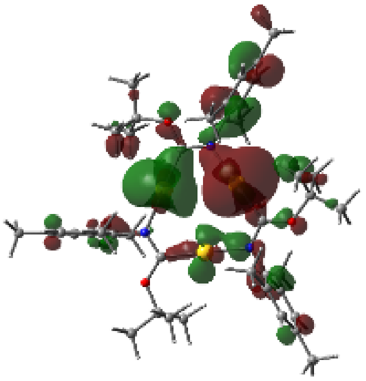	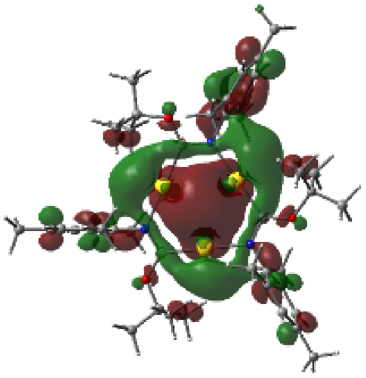
	S_5	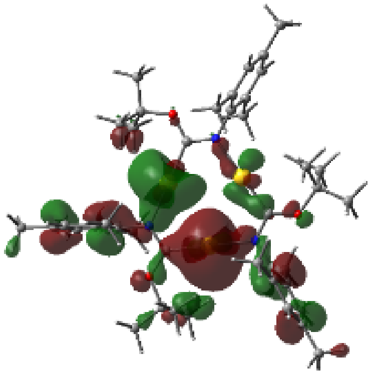	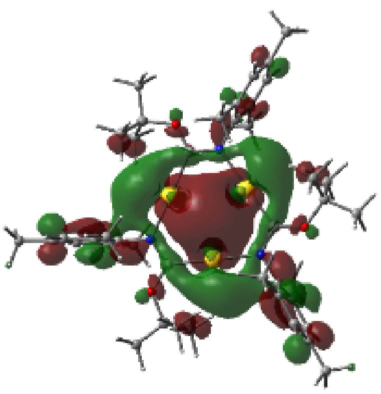

	T_1	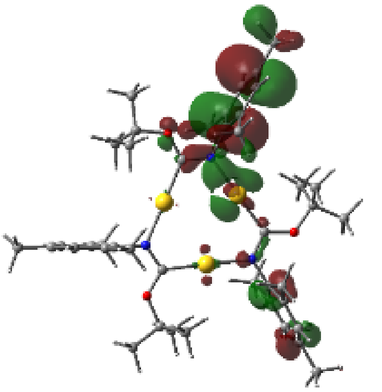	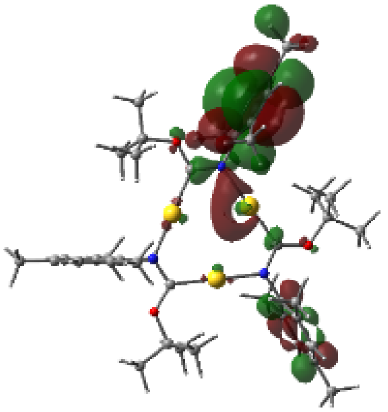
	T_2	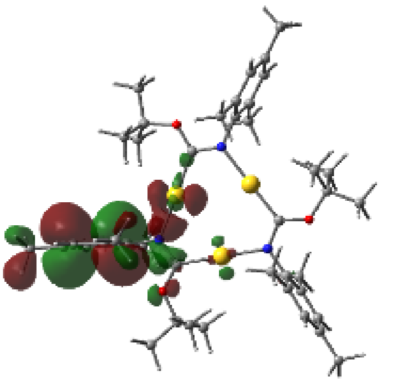	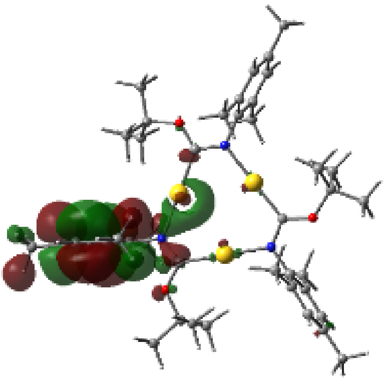
	T_3	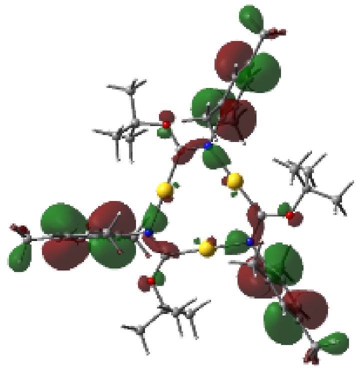	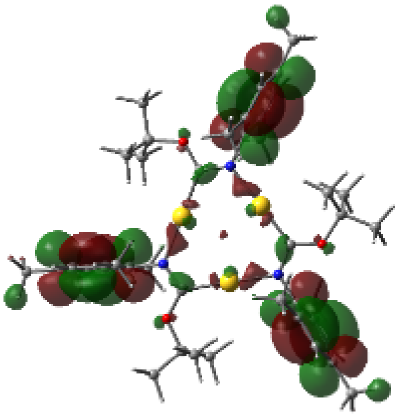
